# Single-cell RNA sequencing reveals the developmental program underlying proximal–distal patterning of the human lung at the embryonic stage

**DOI:** 10.1038/s41422-023-00802-6

**Published:** 2023-04-21

**Authors:** Shangtao Cao, Huijian Feng, Hongyan Yi, Mengjie Pan, Lihui Lin, Yao Santo Zhang, Ziyu Feng, Weifang Liang, Baomei Cai, Qi Li, Zhi Xiong, Qingmei Shen, Minjing Ke, Xing Zhao, Huilin Chen, Qina He, Mingwei Min, Quanyou Cai, He Liu, Jie Wang, Duanqing Pei, Jiekai Chen, Yanlin Ma

**Affiliations:** 1 Guangzhou Laboratory, Guangzhou, Guangdong China; 2 Hainan Provincial Key Laboratory for Human Reproductive Medicine and Genetic Research, Hainan Provincial Clinical Research Center for Thalassemia, Reproductive Medical Center, International Technology Cooperation Base “China-Myanmar Joint Research Center for Prevention and Treatment of Regional Major Disease” By the Ministry of Science and Technology of China, The First Affiliated Hospital of Hainan Medical University, Hainan Medical University, Haikou, Hainan China; 3 Center for Cell Lineage and Atlas (CCLA), Bioland Laboratory (Guangzhou Regenerative Medicine and Health Guangdong Laboratory), Guangzhou, Guangdong China; 4 The Fifth Affiliated Hospital of Guangzhou Medical University, Guangzhou, Guangdong China; 5 CAS Key Laboratory of Regenerative Biology, Guangzhou Institutes of Biomedicine and Health, Chinese Academy of Sciences, Guangzhou, Guangdong China; 6 University of the Chinese Academy of Sciences, Beijing, China; 7 Laboratory of Cell Fate Control, School of Life Sciences, Westlake University, Hangzhou, Zhejiang China; 8 Guangdong Provincial Key Laboratory of Stem Cell and Regenerative Medicine, Guangzhou Institutes of Biomedicine and Health, Chinese Academy of Sciences, Guangzhou, Guangdong China; 9 Key Laboratory of the Ministry of Education for Reproductive Health Diseases Research and Translation, Hainan Medical University, Haikou, Hainan China; 10 Centre for Regenerative Medicine and Health, Hong Kong Institute of Science & Innovation, Chinese Academy of Sciences, Hong Kong, China

**Keywords:** Developmental biology, Stem-cell differentiation

## Abstract

The lung is the primary respiratory organ in human, in which the proximal airway and the distal alveoli are responsible for air conduction and gas exchange, respectively. However, the regulation of proximal–distal patterning at the embryonic stage of human lung development is largely unknown. Here we investigated the early lung development of human embryos at weeks 4–8 post fertilization (Carnegie stages 12–21) using single-cell RNA sequencing, and obtained a transcriptomic atlas of 169,686 cells. We observed discernible gene expression patterns of proximal and distal epithelia at week 4, upon the initiation of lung organogenesis. Moreover, we identified novel transcriptional regulators of the patterning of proximal (e.g., THRB and EGR3) and distal (e.g., ETV1 and SOX6) epithelia. Further dissection revealed various stromal cell populations, including an early-embryonic BDNF^+^ population, providing a proximal–distal patterning niche with spatial specificity. In addition, we elucidated the cell fate bifurcation and maturation of airway and vascular smooth muscle progenitor cells at the early stage of lung development. Together, our study expands the scope of human lung developmental biology at early embryonic stages. The discovery of intrinsic transcriptional regulators and novel niche providers deepens the understanding of epithelial proximal–distal patterning in human lung development, opening up new avenues for regenerative medicine.

## Introduction

As the primary respiratory organ, the lung is essential for terrestrial vertebrates to fuel aerobic metabolism. Pulmonary development is a successive branching morphogenesis process orchestrated by intrinsic molecular machinery and microenvironment. Investigating the mechanism of lung organogenesis facilitates tissue engineering and regenerative medicine, including niche reconstruction for respiratory diseases.

The lung develops at about 4 weeks of gestation in human as a ventral outgrowth of the foregut endoderm surrounded by mesoderm.^1^ Morphologically, the development program undergoes five distinct stages: embryonic (4–7 weeks), pseudoglandular (5–17 weeks), canalicular (16–26 weeks), saccular (26–38 weeks), and alveolar (36 weeks–3 years).^2–4^ During this process, the lung endoderm progenitors differentiate into proximal airway epithelial cell types, such as ciliated, basal, and secretory cells, as well as the distal alveolar cell types.^1,5^ The determination of the proximal–distal pattern is essential for lung morphogenesis. During the pseudoglandular stage of mouse lung development, Sox2^+^ and Sox9^+^ cells are predominantly localized in the proximal and distal epithelium, respectively.^6^ However, in human lung development, the proximal epithelium expresses SOX2, whereas the distal epithelium co-expresses SOX2 and SOX9,^7^ indicating developmental divergence across species. Recently, cell heterogeneity of pseudoglandular to adult lung has been identified at the single-cell level (Supplementary information, Fig. S1a),^8–18^ revealing differentiation details of the airway and alveolar cells. However, how the proximal–distal patterning initiates at early stages (i.e., embryonic and early pseudoglandular stages) in human remains unclear.

Here we performed single-cell RNA sequencing (scRNA-seq) for human embryonic lungs at 4–8 weeks post fertilization. We identified novel cell type-specific markers, including embryonic epithelial transcription factors (TFs), and mapped niche interactome among organogenesis-initiation cell types. These analyses would provide a vital link between gastrulation and fetal lung development, deepen the understanding of human lung biology and spur regenerative medicine research.

## Results

### The major components required for lung development are readily available at the initiation of lung formation

**Fig. 1:**
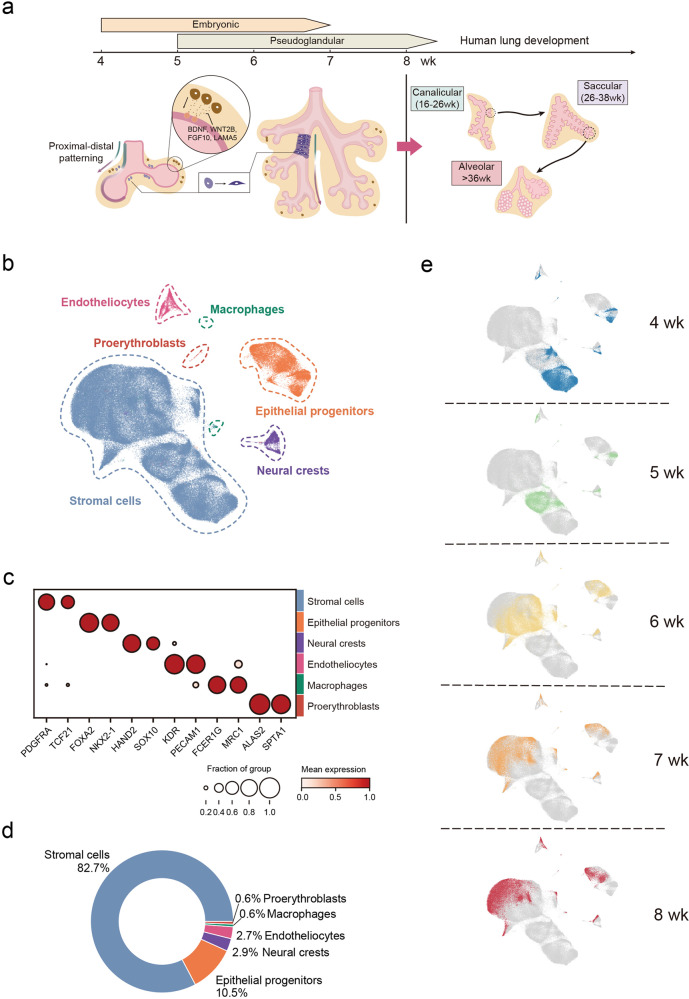
Major pulmonary cell types emerge at the initiation of human embryonic lung development. **a** Schematic overview of scRNA-seq experimental design focusing on embryonic and pseudoglandular stages of human lung development. Sample images are presented in Supplementary information. Fig. S1b. **b** t-SNE projection visualizing 169,686 human embryonic lung cells, clustered into six major cell types. **c** Dot plot showing the top 2 cell type marker expression. The size and color of each dot represent the expression percentage and average expression of the indicated gene in each cell type, respectively. **d** Pie plot showing the proportions of six major cell types. **e** t-SNE layout mapping the distribution of single-cell profiles of each time point denoted as the embryonic week (wk).

Anatomically, the human embryonic lung emerges from the foregut at the beginning of the fourth week post fertilization as tracheal buds, subsequently generating the left and right lung lobes.^4,6,7^ This early stage of human lung organogenesis has rarely been described at the cellular level. Here we intensively sampled human embryonic lungs from Carnegie stage (CS) 12 to 21 (embryonic weeks 4–8), covering both embryonic and early pseudoglandular stages (Fig. 1a and Supplementary information, Fig. S1b). Using 10× Genomics Chromium platform, we obtained high-quality scRNA-seq profiles from these organs, consisting of 169,686 cells with an average of 3764 detected genes and 12,118 unique molecular identifier (UMI) counts (Supplementary information, Fig. S1c). Principal component analysis (PCA) of pseudo-bulk RNA-seq showed that gene expression profiles were arranged by their sampling time, demonstrating a gradual molecular change during development (Supplementary information, Fig. S1d). We annotated six human embryonic lung cell clusters, including lung stromal cells, epithelial progenitors, neural crest progenitors, endothelial progenitors, proerythroblasts, and macrophages, according to their corresponding developmental signatures (Fig. 1b–d and Supplementary information, Fig. S1e).

Among these cells, stromal cells occupy the highest percentage throughout the sampling period, followed by epithelial cells, neural crests, and endothelial cells (Fig. 1d). In addition to well-known cell type markers, we discovered several novel cell type-specific markers (Supplementary information, Fig. S2a and Table S1). Single-molecule inexpensive fluorescence in situ hybridization (smiFISH) validated the specific cellular localization of these cell type markers, including *CTNN2*, *SPINT1*, *CRLF1*, and *PERP* in epithelial cells, *ESAM* and *CD93* in endothelial cells, *TSHZ3* in stromal cells, *KIF1A* in neural crest cells, and *ADAP2* in macrophages (Supplementary information, Fig. S2a, b and Table S1). Moreover, we noticed the heterogeneity within each major cluster and further categorized them into 33 subtypes with subtype-specific features (Supplementary information, Fig. S2c, d and Table S2). TOP2A^+^ proliferative cells were identified in all major clusters, representing active cell division during early embryonic development (Supplementary information, Fig. S2c, d). Thus, our dataset captures the cellular architecture of human embryonic lung. Interestingly, we observed that all six cell types emerge as early as week 4 (CS12), revealing a readily assembled machinery and suggesting that lung organogenesis initiates with complex cellular crosstalk (Fig. 1e and Supplementary information, Fig. S1e).

### TFs modulate proximal–distal patterning of epithelial cells

Lung lobes emerge from trachea/foregut region by a budding process that initiates a series of branching morphogenesis on the lung epithelial progenitors. The epithelial cell has been the primary research focus among six primary lung cell types, due to its intrinsic property to undergo branching morphogenesis. We performed an in-depth analysis of the epithelial cluster, the second largest cluster (17,860 cells) to stromal cells.

**Fig. 2:**
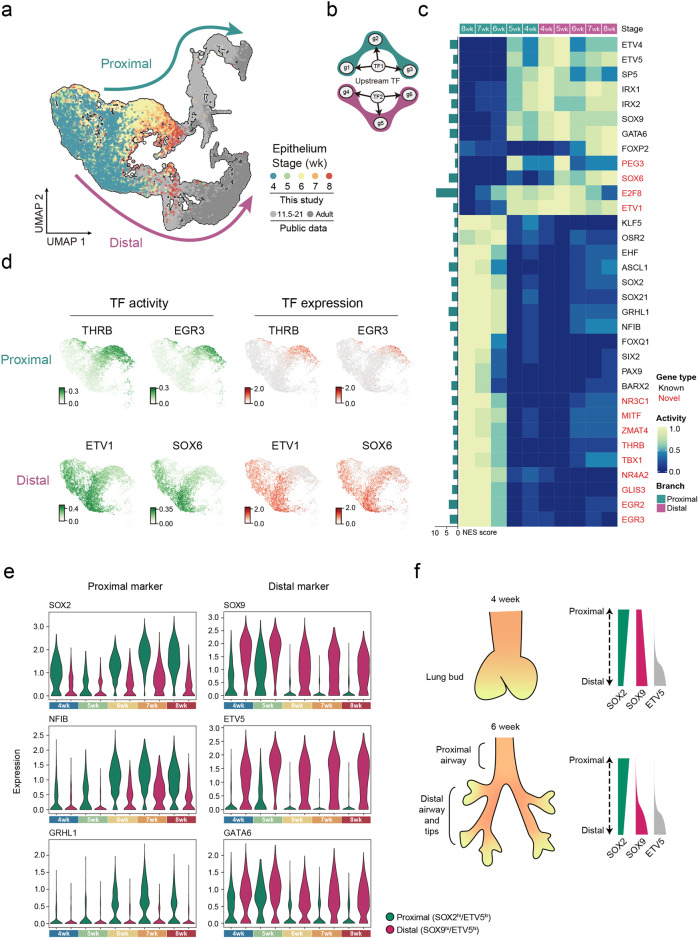
TFs regulate early epithelial proximal–distal patterning. **a** UMAP layout showing the integration of human epithelial scRNA-seq dataset from this study (colored by sample collection time points) and published datasets^9,13^ (colored by gray). Upper (green arrow) and lower (magenta arrow) trajectories represent proximal and distal epithelial lineages, separately. **b** Illustration of TF–gene regulons, inferred by SCENIC. **c** Heatmap showing temporal changes of regulon activity in proximal (green) and distal (magenta) branches. The bar graph on the left represents the Normalized Enrichment Score (NES) of each TF. Genes marked in red are newly identified TFs in this study. **d** TF activity (green) and expression (red) of proximal-specific *THRB* and *EGR3* (above) and distal-specific *ETV1* and *SOX6* (bottom) during weeks 4–8 are projected on UMAP. SCENIC-generated TF activity, represented by AUC score, reflects the co-expression strength of TF and its target genes. **e** Violin plots showing the expression of the *SOX2*, *NFIB*, *GRHL1*, *SOX9*, *ETV5* and *GATA6* in EPI_SOX2^hi^/ETV5^lo^ and EPI_SOX9^hi^/ETV5^hi^ cells from weeks 4–8. **f** Illustrations of the proximal–distal patterning and marker gene expression.

To expand the timeline of single-cell snapshots during lung epithelial differentiation, we integrated our dataset (the earliest time points) with publicly available datasets spanning week 11 to adult^9,13^ (Fig. 2a). This integrated dataset demonstrates that weeks 4–8 serves as a branch point where most progenitors of proximal (e.g., basal and ciliated cells) and distal (e.g., alveolar type 1 and type 2 cells (AT1 and AT2)) trajectories have emerged (Supplementary information, Fig. S3a, b). The heterogeneity of proximal/distal progenitors promoted our investigation of TFs, a major driving force for cell fate decisions during development. We first calculated the TF–gene (regulon) activity during weeks 4–8 using single-cell regulatory network inference and clustering (SCENIC^)19^ (Fig. 2b; see Materials and Methods). Next, Waddington-OT^20^ (WOT) was used to infer the score of proximal and distal cell fates by calculating the cell–cell similarity between each adjacent time points (see Materials and Methods). Finally, we obtained the weighted sum of regulon activities based on WOT trajectory scores at each time point. The identification of known TFs regulating proximal (e.g., KLF5^21^/SOX2^22,23^) and distal (e.g., ETV5^24^/SOX9^25^) cell fates verified this strategy (Fig. 2c). Moreover, we revealed novel proximal (e.g., THRB, MITF, EGR3) and distal (e.g., ETV1, SOX6) TF regulators (Fig. 2c, d and Supplementary information, Table S3). These proximal–distal patterning TFs exhibit trajectory-specific TF activity and expression, serving as potential TFs regulating epithelial cell fates (Fig. 2c). Furthermore, SCENIC also identified *SOX2/NFIB/NCAM1* and *ETV5/EFNB2* as the downstream targets of THRB and ETV1, respectively (Supplementary information, Fig. S3c, d). These proximal (e.g., *SOX2*^22,23^/*NFIB*^26^/*NCAM1*^27^) and distal (e.g., *ETV5*^24^*/EFNB2*^28^) associated targets are reported to contribute to epithelial regionalization, echoing the role of the newly identified factors THRB and ETV1 as proximal–distal patterning regulators. Consistently, we observed the smiFISH co-localization of *SOX9* and the newly identified distal regulators, *ETV1/SOX6*, in the distal epithelium, while *THRB*, the novel proximal regulator, was expressed in the proximal epithelial tissue with positive staining of *NKX2-1* (Supplementary information, Fig. S3e). Our investigation highlighted the molecular signatures of proximal–distal patterning during weeks 4–8, far preceding the first morphological sign of distal epithelial differentiation at the canalicular stage.^4^

Unlike epithelial cells at week 8 with clear segregation along proximal/distal trajectories, cells at early time points remained merged (Fig. 2a), raising the question of how and when the proximal–distal patterning initiates in the human lung epithelium development. The epithelial cells consisted of 5 populations, including subtypes highly expressing known proximal/distal markers, SOX2 (EPI_SOX2^hi^/ETV5^lo^) and SOX9/ETV5 (EPI_SOX9^hi^/ETV5^hi^), representing proximal and distal lineages, respectively (Supplementary information, Figs. S3b and S4a, b and Table S4). To trace the precise origin of the proximal–distal patterning, we analyzed the developmental trajectory of epithelial cells for each time point at weeks 4, 6, and 8 (Supplementary information, Fig. S4c). RNA velocity analysis^29^ suggested that the EPI_SOX2^hi^/ETV5^lo^ and EPI_SOX9^hi^/ETV5^hi^ populations were derived from proliferating subtypes, EPI_DTL^+^ and EPI_TOP2A^+^, indicating lung epithelial progenitors as the source of the prospective proximal/distal epithelium. Furthermore, some EPI_SOX2^hi^/ETV5^lo^ cells were derived from EPI_SOX9^hi^/ETV5^hi^ cells, consistent with the reported evidence that tip progenitors in distal could differentiate into proximal lineages.^4,30,31^ These processes occurred as early as week 4, suggesting that the proximal–distal patterning initiates immediately after lung organogenesis starts (Supplementary information Fig. S4c). Indeed, discernible proximal–distal expression patterns were observed in EPI_SOX2^hi^/ETV5^lo^ and EPI_SOX9^hi^/ETV5^hi^ cells at week 4 (Fig. 2e, f). Notably, SOX2 and SOX9 were co-expressed in both populations until the substantial downregulation of distal factors in EPI_SOX2^hi^/ETV5^lo^ cells since week 6 (Fig. 2e, f), suggesting the initiation of a proximal specification around week 6. Consistently, the transcriptional activity of SOX2 surged at week 6 (Fig. 2c).

We were curious about the conservation of identified TFs in the proximal–distal epithelial patterning. To this end, we performed scRNA-seq of mixed viscera from mouse embryos, sampled at 0.5-day intervals from E12.0 to E14.0. The lung epithelium, denoted by Nkx2-1 and Cdh1 expression, was selected for analysis (Supplementary information, Fig. S5a, b; see Materials and Methods). Proximal and distal epithelial cells were defined by previously reported TFs in mouse (Supplementary information, Fig. S5c). Integrating human and mouse epithelial datasets, we identified shared proximal (e.g., SOX2, OSR2, ASCL1, SOX21, and EHF) and distal (e.g., SOX9, ETV4, ETV5, IRX2, and GATA6) TF regulators, indicating the conserved core transcriptional regulation network in proximal–distal patterning. On the other hand, human- and mouse-specific TFs were also identified (Supplementary information, Fig. S5d and Table S5), illustrating species-specific developmental programs. The putative human development-specific TFs, such as SOX6, ETV1, THRB, and GLIS3 could be valuable for investigating human lung evolution.

Together, our analysis scrutinized early human pulmonary epithelial development. We conclude that the proximal–distal patterning of lung epithelium occurs since week 4 and is oriented by different combinations of TFs.

### Stromal cells contribute to diverse microenvironments for epithelial proximal–distal patterning

In addition to intrinsic TFs, the microenvironment resulting from the specification of mesoderm-derived stromal cells is critical to shaping lung epithelial morphogenesis.^32,33^ The stromal cell cluster is the largest population among pulmonary cell types during weeks 4–8 (Fig. 1d). We thus sought to investigate how these stromal cells influence human lung epithelial development at the embryonic stage.

**Fig. 3:**
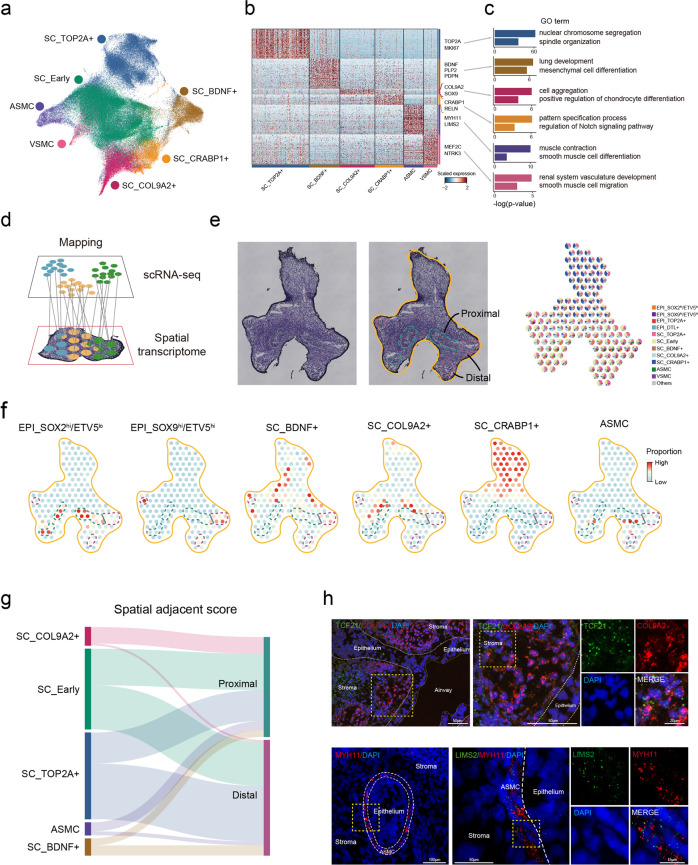
Multiple stromal cell types exhibit proximal–distal spatial heterogeneity. **a** Seven stromal cell subtypes were identified based on Leiden clustering (*r* = 0.3). **b** Heatmap showing differentially expressed genes (DEGs) of six stromal cell subtypes, with no DEGs for SC_Early (Wilcoxon rank-sum test, *P* value < 0.01). **c** Bar plots showing the GO terms enriched in six stromal cell subtypes. GO enrichment was performed by clusterProfiler. **d** Illustration of mapping single-cell transcriptome data to spatial transcriptome data (10× Visium) using Tangram algorithm. **e** Frozen section of a 6-week human lung (left panel). The illustration in the middle panel shows the outline of lung (colored by orange), the proximal epithelium (colored by green) and the distal epithelium (colored by magenta). Pie plots show the proportion of cell types mapped to each Visium spot from single-cell transcriptome data (right panel). **f** Dot plots showing the proportion of two epithelial cell types and four stromal cell types mapped to 10× Visium spots. The colored dash lines highlight the proximal and distal regions as illustrated in **e**. **g** Sankey plot showing the spatial adjacency of stromal cell types and two epithelial cell types. The line indicates the proportion of stromal subtypes in the epithelium-located spots in the proximal or distal region and the surrounding spots, with thicker lines indicating more stromal cells adjacent to the proximal or distal epithelium, and vice versa (Supplementary information, Table S11; see Materials and Methods). The absence of a connection between stromal and epithelial subtypes means no proximity. **h** smiFISH showing the expression of *COL9A2* (red) and *TCF21* (green) in SC_ COL9A2^+^ along the bronchus, and *LIMS2* (green) and *MYH11* (red) in ASMC surrounding epithelial cells in the lung at week 8. Data are representative of at least three independent smiFISH experiments.

We focused on the heterogeneity of stromal cells and their contribution to epithelial development. We identified 7 subtypes of stromal cells, including five stromal subtypes and two smooth muscle cell (SMC) subtypes (i.e., airway SMC (ASMC) and vascular SMC (VSMC)) according to their molecular signatures (Fig. 3a and Supplementary information, Fig. S2c, d). Notably, the heterogeneity of the early-stage stromal population was previously underestimated due to the lack of single-cell investigations. Among five stromal subtypes, SC_TOP2A^+^ represents proliferating stromal cells. Additionally, we newly defined SC_COL9A2^+^, SC_CRABP1^+^, and SC_BDNF^+^ for further characterization (Fig. 3a, b). SC_COL9A2^+^ enriches the GO term of positive regulation of cartilage development, indicating a cricoid cartilage fate encircling the trachea.^34^ SC_CRABP1^+^ is a subtype regulating Notch signaling. SC_BDNF^+^ is related to mesenchymal differentiation and lung development (Fig. 3c and Supplementary information, Table S4).

To further resolve the spatial properties of these stromal cells, especially, their proximity to epithelium, we performed spatial transcriptomic profiling of the 6-week lung tissue sections by 10× Visium technique. Tangram algorithm was applied to integrate single-cell transcriptome with the spatial data, mapping molecular features to the anatomical scale^35^ (Fig. 3d). As a proof of principle, we observed the distribution of epithelial subtypes, EPI_SOX2^hi^/ETV5^lo^ and EPI_SOX9^hi^/ETV5^hi^, along with their molecular markers, in the proximal and distal epithelium, respectively (Fig. 3e, f and Supplementary information, Fig. S6a, b). Moreover, an additional tissue section was examined, which exhibited a similar cell type proportion and signature expression pattern, demonstrating a minimal batch effect (Supplementary information, Fig. S6c–e). We then calculated the spatial proximity of stromal subtypes to the proximal/distal epithelium, as illustrated by Sankey plot (Fig. 3g; see Materials and Methods). Accordingly, we observed that SC_CRABP1^+^ intensively surrounded the tracheal tube. SC_COL9A2^+^ located along the left and right bronchi. In contrast, SC_BDNF^+^ resided mainly in the border region, implying a critical role in establishing the distal epithelial niche. ASMC was distributed around the epithelium, whereas VSMC was scattered without any typical pattern. smiFISH validated the spatial locations of SC_COL9A2^+^ and ASMC with markers *COL9A2/TCF21* and *MYH11/LIMS2*, respectively (Fig. 3h). Notably, *LIMS2* is a newly identified ASMC marker in this study. These results demonstrate the specificity of stromal subtype distribution and their diverse roles as niche providers in epithelial morphogenesis during embryonic lung organogenesis.

**Fig. 4:**
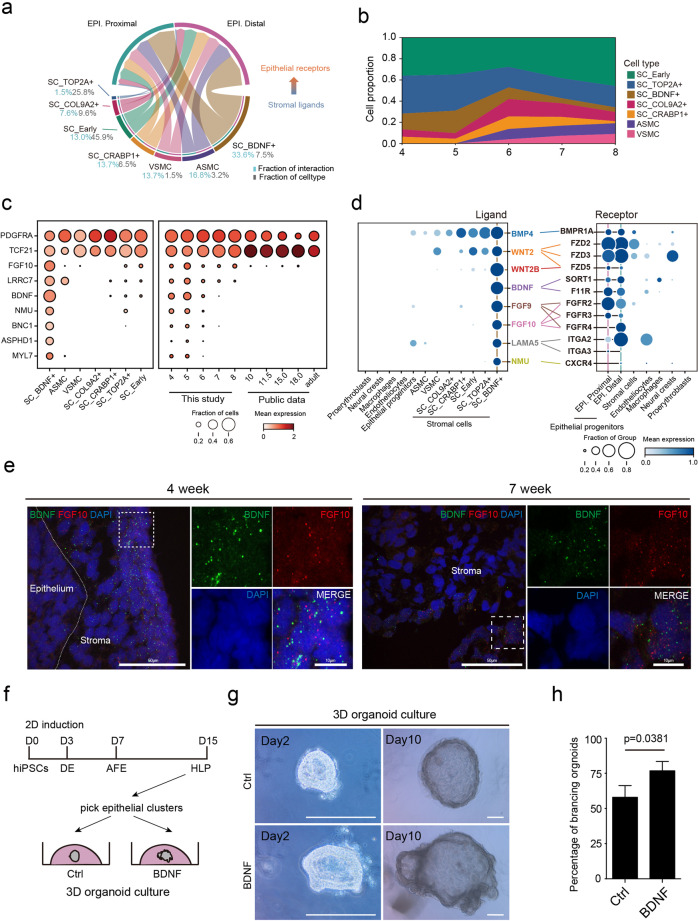
SC_BDNF^+^ stromal cells provide signals for early lung epithelial development. **a** Circos plot illustrating significant ligand–receptor interactions among stromal cells and epithelial cells, determined by permutation test (see Materials and Methods). The percentage in gray represents the proportion of ligand–receptor pairs of each stromal subtype; the percentage in blue represents the proportion of each stromal subtype in all stromal cells. EPI epithelial progenitors. **b** Stack plot showing the cell proportion of seven stromal subtypes during weeks 4–8. **c** Dot plot showing the specific gene expression profile of SC_BDNF^+^ across stromal cell types (left frame) and developmental stages (right frame; weeks 4–8, this study; after week 10, previous studies^9,13,18^). The size and color of each dot represent the expression percentage and expression level of the indicated marker gene within each cell type (left frame) and at each time point (right frame), respectively. **d** Dot plot showing the expression percentage and level of ligands (left) and receptors (right) among stromal and epithelial cell types, which highlights a high ligand–receptor interaction between SC_BDNF^+^ and epithelial cells. Colored lines connect ligand–receptor pairs. **e** smiFISH (left) showing the expression of *BDNF* and *FGF10* in a group of stromal cells located around the epithelial cells and in the border region of the lung at week 4 while only in the border region at week 7. Data are representative of at least two independent smiFISH experiments. Scale bars, 50 μm (long), 10 μm (short). **f** Schematic diagram showing human lung epithelial organoids generated from hiPSCs and used for the validation of BDNF effects. DE definitive endoderm, AFE anterior foregut endoderm, HLP human lung progenitors. **g** Representative morphologies showing that BDNF promotes human lung organoid branching compared with the control group. Data are representative of 10–15 organoids from each of three independent experiments. Scale bars, 250 μm. **h** Quantification of the percentages of branching organoids upon the addition of BDNF. Data are means ± SD, *n* = 3 independent experiments. Unpaired Student’s *t*-test, *P* < 0.05.

To investigate the niche for early lung development, we performed ligand–receptor interaction analysis among major lung cell types (Supplementary information, Fig. S7a and Table S6; see Materials and Methods). Specifically, stromal cells and endotheliocytes display the highest level of connectivity with epithelial cells. Previously reported niche factors modulating epithelial development, including WNT,^36^ FGF,^37–39^ and BMP^40,41^ signals, are derived explicitly from stromal cells (Supplementary information, Fig. S7b), reflecting a stromal-derived microenvironment orchestrating airway branching morphogenesis. We further analyzed the contribution of stromal subtypes to the epithelial niche. Interestingly, we observed that the BDNF^+^ stromal subtype, consisting of only 7.5% stromal cells, was responsible for > 33% stromal-derived ligands for epithelial receptors (Fig. 4a and Supplementary information, Table S6). It is of interest that the BDNF^+^ stromal subtype appeared at week 4 and gradually exhibited a decreased cell proportion along lung development (Fig. 4b). Meanwhile, signature genes of this cell type, including BDNF and FGF10, were also downregulated during organogenesis (Fig. 4c), highlighting the unique role of this BDNF^+^ cell population at the early embryonic stage. Ligand–receptor interaction analysis further elucidated that the BDNF^+^ stromal subtype primarily accounts for the expression of ligands, including BDNF, FGF9/10, WNT2/2B, LAMA5, and NMU, whose receptors are epithelial-dominant, demonstrating highly specific BDNF^+^ stromal–epithelial communication (Fig. 4d). GO terms related to signatures of this subtype encompass mesenchymal differentiation and lung development (Fig. 3c). By multiplying the ligand and receptor expression levels in cell types mapped to each spot and its neighboring spots, we inferred that BNDF^+^ cells-derived ligands interacted with the receptors on epithelial cells (Supplementary information, Fig. S7c). Notably, BMP4, WNT2/2B, FGF9, and FGF10 signaling are known players in early lung specification and branching morphogenesis.^36–46^ These analyses emphasized that the BDNF^+^ stromal subtype is critical for early-stage epithelial development by secreting abundant growth factors.

Consistent with the predicted spatial distribution mentioned above, smiFISH showed the presence of BDNF^+^/FGF10^+^ stromal cells expressing high-level BDNF/FGF10 at the margin of developing lungs at week 4; and the expression levels of BDNF/FGF10 decreased at week 7 (Fig. 4e). Though BDNF^+^ cells showed mesenchymal morphology, they shared several signatures (e.g., WT1) with the annotated mesothelium in mouse, a squamous epithelial cell type covering the lung at later developmental stages^47^ (Supplementary information, Fig. S8a–c), suggesting that this BDNF^+^ subtype might be the stromal progenitor of the mesothelium. However, the proportions of cells expressing non-mesothelium genes (e.g., BDNF/FGF10/LRRC7) were gradually decreased along the early lung development (Fig. 4c and Supplementary information, Fig. S8c), emphasizing the special role of the BDNF^+^ stromal subtype as an early stage-specific epithelial niche. To examine the role of BDNF as an epithelial niche factor, we supplemented BDNF on 3D lung epithelial progenitor cell (LEPC) organoids derived from human induced pluripotent stem cells (hiPSCs) (Fig. 4f). During daily cell culture, we observed that addition of BDNF improved the survival, growth and branching of LEPC organoids (Fig. 4g, h; see Materials and Methods).

Together, these results demonstrate that SC_BDNF^+^, an embryonic stage-specific subtype, contributes to epithelial branching morphogenesis in early lung development.

### Signatures of stromal cell-derived SMC fate bifurcation

Apart from the BDNF^+^ stromal cell subcluster described above, stromal cell-derived VSMC and ASMC are pivotal for modulating pulmonary function. Ligand–receptor interaction analysis showed that VSMC and ASMC together contributed to over 30% of stromal-epithelial cell crosstalk, indicating their critical roles in lung epithelial development (Fig. 4a). However, the program encoding the developmental origin and cell specification of VSMC and ASMC is unclear. Thus, we explored the developmental program of SMC and its roles in epithelial development.

**Fig. 5:**
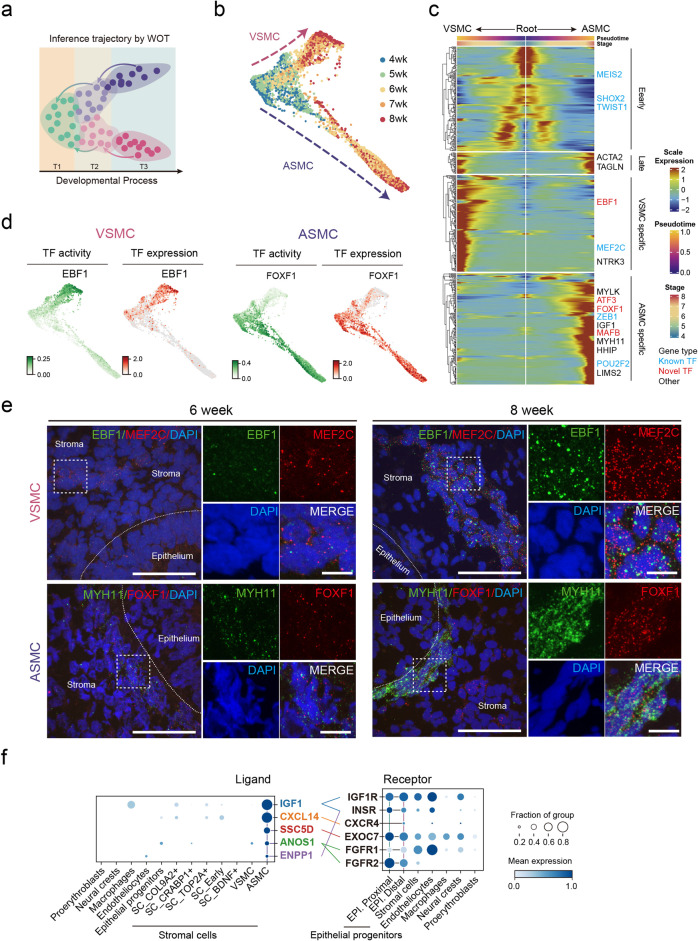
The developmental trajectory inferences of ASMC and VSMC. **a** Schematic diagram of WOT, an approach for trajectory inference using time-course information. **b** Force-directed layout embedding (FLE) to visualize the WOT-inferred VSMC and ASMC developmental trajectories, colored by time points. Upper (red arrow) and lower (purple arrow) trajectories represent VSMC and ASMC lineages, respectively. **c** Heatmap showing the dynamics of gene expression along Palantir pseudotime of ASMC and VSMC development. Genes marked in blue are known TFs, and those in red are newly identified TFs in this study. **d** TF activity and expression of VSMC-specific EBF1 (left) and ASMC-specific FOXF1 (right) were projected on FLE layout. TF activity reflects the co-expression strength of TF and its target genes. **e** smiFISH showing the expression of *EBF1* (green) and *MEF2C* (red) in VSMC and *MYH11* (green) and *FOXF1* (red) in ASMC in the lung at weeks 6 and 8, respectively. Data are representative of at least two independent smiFISH experiments. Scale bars, 50 μm (long), 10 μm (short). **f** Dot plots showing the expression percentage and level of ligands (left) and receptors (right) between the providers (i.e., stromal cells including ASMC) and the recipients (i.e., epithelial cells). Colored lines connect ligand–receptor pairs.

As the SMCs can be defined at week 6 (Fig. 4b), we implemented WOT^20^ algorithm to infer the origin of SMCs at earlier time points (Fig. 5a and Supplementary information, Fig. S9a). Single-cell profiles across time points (weeks 4–6) were individually displayed in UMAP, and the trajectory scores of ASMC and VSMC were colored in blue and green, respectively (Supplementary information, Fig. S9b). Interestingly, WOT inferred that the cell fate of some stromal cells was already biased towards ASMC or VSMC at weeks 4–5, earlier than the stages when ASMC (week 5)^48^ and VSMC (week 7)^49^ were observed anatomically (Supplementary information, Fig. S9c). At week 4, SMC (*ACTA2*,^50,51^ *ACTG2*^52^), ASMC (*MYH11*,^53,54^ *IGF1*,^55,56^ *FHL2*^57^) and VSMC (*MEF2C*,^58^ *EGFL6*,^54,59^ *HEYL*^60^)-specific markers were detected in SMC progenitors, albeit at relatively low levels (Supplementary information, Fig. S9d). These results highlight the cell fate specification of SMC at the embryonic stage of lung development.

Subsequently, cells with trajectory score > 0.0001 were selected as SMC lineage. Force-directed layout showed a clear bifurcation of ASMC and VSMC development (Fig. 5b). By arranging cells along the Palantir^61^ pseudotime (Fig. 5c), we showed that the core TFs of mesenchymal cells, e.g., *MEIS2, SHOX2, TWIST1*, were gradually downregulated with the development of both types of SMCs, whereas the bona fide SMC markers, *ACTA2* and *TAGLN*, were expressed and progressively upregulated in both ASMC and VSMC populations. The expression levels of the reported VSMC and ASMC markers, *MEF2C* and *MYH11*, were specifically upregulated along their corresponding trajectories.^54,58,62^

Next, following the developmental trajectory, we searched for TFs that regulate the differentiation of the two types of SMC. We identified the classical regulators, MEF2C^58,62^ for VSMC and ZEB1^63^ for ASMC, respectively, verifying the robustness of this workflow (Fig. 5c). Accordingly, we uncovered novel TFs for SMC development, i.e., EBF1 for VSMC, and FOXF1 for ASMC. These TFs exhibit high regulon activity and specific transcription patterns (Fig. 5d). Notably, the EBF1 target genes, *NTRK3*^54^ and *PDGFRB*^64^ are unique to VSMC differentiation, whereas *MYH11*^53,54^ and *HHIP*^65^ regulated by FOXF1 are specifically expressed in the ASMC lineage (Supplementary information, Fig. S10a, b and Table S7). These results emphasized the SMC lineage specificity of EBF1 and FOXF1, suggesting their roles in VSMC and ASMC cell fate determination, respectively. To validate this finding, we performed smiFISH experiments to examine the expression patterns of the identified VSMC (i.e., *MEF2C/EBF1/HEYL*) and ASMC (i.e., *MYH11/FOXF1*) markers in lung tissues at weeks 6 and 8 (Fig. 5e and Supplementary information, Fig. S10c). The results showed that VSMC markers were distributed dispersedly at week 6 but appeared obviously in the vascular media at week 8. Meanwhile, ASMC markers were expressed around epithelial cells since week 6. These results further confirmed the predicted spatial and molecular specificity of SMC subtypes during early human lung development.

As ASMC is spatially adjacent to epithelial cells (Fig. 3g), we explored ASMC-specific niche factors modulating epithelial development. ASMC specifically expressed IGF, CXCL14, SSC5D and ENPP as ligands interacting with epithelial receptors (Fig. 5f). Importantly, IGF1 (insulin-like growth factor 1) has been reported to regulate alveolar separation^66^ and basal cell differentiation.^67^ Our data revealed that IGF1 expression is significantly higher in ASMC than in other cells, indicating the essential role of ASMC in lung development. Together, the identification of these SMC-expressing ligands that establish the microenvironment for epithelial development would help improve the generation of in vitro lung organoid models.

## Discussion

In this study, we report a single-cell transcriptome atlas of the human embryonic lung that comprises ~170,000 cells from CS12 to CS21. We characterized 6 major cell clusters which were further divided into 33 cell subtypes, and identified a series of novel cell type-specific signatures. More importantly, taking advantage of this dataset, we systematically investigated the human lung proximal–distal patterning and found that it occurred as early as week 4 upon the initiation of lung organogenesis. Based on the transcriptome-regulon analysis, we identified several novel TFs responsible for driving the proximal–distal patterning. Furthermore, we discovered a BDNF^+^ population as a new embryonic stromal cell subtype producing abundant niche factors such as BDNF, FGF10, WNT2B, and LAMA5. Finally, we deciphered developmental programs and signatures of two distinct SMC subtypes, VSMC and ASMC, and revealed their roles in establishing the microenvironment for epithelial morphogenesis. This study provides a useful resource for lung organogenesis research and regenerative medicine, especially stem cell-based strategies.

Notably, we observed the presence of endoderm-derived epithelial progenitors and mesoderm/ectoderm-derived cell types at the onset of lung development. The rich diversity of cell origins in early lung development suggests the orchestration of different cell types from three germ layers upon organogenesis and proximal–distal patterning. Recently, human lung cell atlases from pseudoglandular to saccular stages were characterized at single-cell resolution^8,9,15,16^ (Supplementary information, Fig. S1a). However, due to the insufficient cell coverage and detected genes per cell, key early embryonic developmental events may be missed in their study. Moreover, the atlas starting from week 4 would serve as a critical benchmark for future modeling of lung organogenesis. Our discovery of early-stage cell types, such as niche and progenitor cells, could optimize the current protocol for in vitro organoid generation. Novel progenitor cells identified from this atlas could facilitate transplantation technologies to treat newborns with lung defects or adults with severe respiratory injury caused by diseases such as COVID-19.

## Materials and methods

### Human embryonic sample collection and dissection

Human embryonic lung samples generated from legal elective abortions were obtained from the First Affiliated Hospital of Hainan Medical University, under a protocol approved by the ethics committee of the First Affiliated Hospital of Hainan Medical University (2017-KY-001) with informed consent obtained from all participants. Embryonic stages were determined based on established morphological landmarks by CS and the gestational time. Samples used in this study were from CS12 to CS23. Embryos were isolated and rinsed in PBS and then transferred into a new dish with PBS to dissect embryonic lung samples. Dissected lung tissues were dissociated into single cells or embedded into optimal cutting temperature (OCT) compound and frozen at –80 °C for further processing.

### Human lung tissue dissociation and single-cell suspension preparation

After verification of period-specific characteristics by microscopy and sample dissection referencing CS tables, human lungs from aborted embryos of defined stages were separated into LoBind centrifuge tubes (Eppendorf, 0030108310) with basal medium (RPMI-1640 medium:DMEM/F12 = 1:1). Then pre-cooled PBS was used to wash the tissues twice. The embryonic lungs were digested by preheated 10 U/mL Papain (Worthington, LS003126) at 37 °C for 20 min in the incubator whilst the time would be prolonged to 30 min for the sample from later periods. Proper agitation was needed to promote digestion every 10 min. Once the digestion was completed, an equivalent 10% FBS was added for termination and 100 U/mL DNase I was used along by pipetting repeatedly. Cell suspensions were filtered through a 40-μm strainer (Falcon, 352340) and collected into a new LoBind tube on ice. After centrifugation at 300× *g* for 5 min at 4 °C, the cell pellets were treated with 1 mL of ACK lysis buffer (Gibco, A1049201) to remove erythrocytes for 2 min at room temperature and then triple volume of PBS was added to quench the lysis reaction. Subsequently, cell suspension was centrifuged at 300× *g*, 4 °C for 5 min, then washed twice and resuspended with PBS plus 0.04% BSA to prepare for scRNA-seq library construction. Meanwhile, 0.04% trypan blue was introduced to determine cell viability via cell staining.

### Mouse embryo dissociation and single-cell suspension preparation

Embryos from E12.0 to E14.0 were washed with cold PBS twice and dissected to collect visceral organs for single-cell suspension. The viscera were digested in 1 mg/mL collagenase I/II/IV for 30 min at 37 °C, 5% CO_2_ and pipetted gently every 10 min. After digestion, cells were filtered through 40-µm cell strainers on ice and centrifuged at 4 °C, 300× *g* for 5 min. Cells were treated with ACK lysis buffer for 5 min at room temperature to remove red blood cells and washed once with cold PBS with 0.04% BSA. Trypan blue staining solution was added to count cell numbers and assess cell viability. Cells were then collected in PCR tubes for single-cell library preparation and sequencing. For downstream single-cell analysis, mouse lung epithelial cells were selected based on a group of traditionally known markers like Nkx2-1/Cdh1.

### Single-cell RNA library preparation and sequencing

Single-cell suspensions from each sample were loaded onto 10× Genomics Chromium v3.1 system to generate single-cell gel beads-in-emulsion (GEMs), where all generated full-length cDNA share a common 10× barcode. After incubation, GEMs were disrupted and cDNA was amplified via PCR. The single-cell 3’ gene expression libraries were constructed using 10 μL (a proportion of 25%) of the total cDNA and purified with SPRIselect. Libraries were quality controlled by Qsep100 for sized distribution and by Qubit 4.0 fluorometer for concentration quantification. Finally, sequencing was performed on Illumina NovaSeq system with 200G paired bases in PE150 mode.

### Spatial transcriptomic library preparation and sequencing

Spatial transcriptomics analysis for human developing lungs was performed using the 10× Genomics Visium Spatial Gene Expression assay kit according to the manufacturer’s recommendations. Briefly, the embryonic lung tissue was incubated in OCT at 4 °C and then frozen in OCT and stored at –80 °C. The embedded lung tissues were cut into cross sections with a thickness of 10 μm and placed onto a Visium Gateway gene expression slide. Before library construction, the permeabilization time for spatial transcriptomics assay was optimized to be 18 min using the Visium Spatial Tissue Optimization slide by GENE DENOVO. Haematoxylin and eosin staining was performed and imaged using a Nikon microscope. Accordingly, embryonic lung tissue sections were permeabilized to capture RNA molecules with barcoded spots on the Visium slides. Libraries were prepared and quality controlled by Agilent 2100 for sized distribution and by Qubit 4.0 fluorometer for concentration quantification. Libraries were sequenced on MGISEQ2000.

### Tissue cryosection preparation

Human lung tissues were surgically stripped and washed with PBS, then embedded in Tissue-Tek OCT compound at 4 °C for 30 min, before freezing at –20 °C. One hour later, the samples were transferred to a –80 °C low-temperature freezer and stored for a long time. The embedded lung tissues were cut into cross sections with a thickness of 10 μm in cryostat for smiFISH.

### smiFISH

#### Tissue slice preparation and fixation

The 10-μm sections were collected using a Leica CM3050S cryostat at –20 °C with adhesion microscope slides. The slide-mounted sections were fixed for 10 min at room temperature in 4% paraformaldehyde in PBS, washed three times with PBS for 5 min, then immersed in 70% (vol./vol.) ethanol for at least 1 h at room temperature. Slides can be stored at 2–8 °C in 70% ethanol up to a week before hybridization.

#### Probe preparation

The smiFISH primary probes and FLAPs (secondary probes with fluorescence) were produced as previously described.^68^ The primary probes were synthesized and purchased from Tsingke Biotechnology, China. The secondary probes were conjugated to one Cy3/Cy5 moiety through 5’ amino modifications, and purchased from Thermo Fisher Scientific, China. All probe sequences are available in Supplementary information, Table S8.

#### Hybridization or immunofluorescence co-staining

The fixed slide-mounted sections were circumscribed with a PAP pen and slides were washed by Wash Buffer A (Biosearch Technologies, USA) for 2–5 min. Slides were subsequently incubated in Hybridization Buffer (Biosearch Technologies, USA) containing 125 nM probes (with primary antibodies) in an opaque humidity chamber at 37 °C, overnight. Pre-warmed Wash Buffer A (with second antibodies) was used to wash the slides twice for 30 min each at 37 °C. Slides were incubated with 200 ng/mL DAPI in Wash Buffer B (Biosearch Technologies, USA) for 5 min to counterstain nuclei, and then washed again with Wash Buffer B. Slides were then mounted using a minimal volume (~50–100 μL) of the mounting medium onto the tissue section, and covered with a clean cover glass. Clear nail polish was allowed to seal the cover glass perimeter.

#### Microscopy

Images were captured using a Dragonfly 200 High-Speed Confocal Imaging Platform (Andor, USA), consisting of an Andor iXon Ultra EMCCD and a Nikon Eclipse Ti2-E Inverted Microscope. Image capture or processing was done using Andor Fusion Software, Adobe Photoshop CC 2018, and Fiji in ImageJ, with brightness, contrast, pseudo-coloring adjustments, and *z*-stack alignments applied equally across all images in a given series.

### Human lung epithelial organoid generation

hiPSCs were generated from human urine cells in our lab and cultured in mTeSR1 medium in 24-well plates coated with Matrigel. When the cell confluence achieved 80%–90%, hiPSCs were treated with definitive endoderm (DE) medium containing RMPI1640 supplemented with 100 ng/mL Activin A (Peprotech, 12014500), 5 μM Y27632 (TargetMoI, T1870) and 2 μM CHIR99021 (TargetMoI, T2310) for 3 days. Then, the medium was changed into anterior foregut endoderm (AFE) medium including basal medium DMEM/F12 with 100× N2, 50× B27, 50 ng/mL Vc (Sigma-Aldrich, 49752), 0.4 mM MTG (Sigma-Aldrich, 6145), 500 ng/mL FGF4 (Peprotech, 100-31), 1 μM SAG (Selleck, 6384), 10 μM SB431542 (TargetMoI, T1726), 200 ng/mL Noggin (Peprotech, 120-10C) for 2 days; and Noggin was later replaced with 1 μM IWP-2 (TargetMoI, T2702) for another 2 days. Afterward, cell fates were further induced by human lung progenitor (HLP) cell medium with DMEM/F12 with 100× N2, 50× B27, 50 ng/mL Vc, 0.4 mM MTG, 20 ng/mL BMP4 (R&D, 314-BP), 10 ng/mL FGF7 (Peprotech, 100-19), 10 ng/mL FGF10 (Peprotech, 100-26), 3 μM CHIR99021, 0.1 μM RA (Selleck, NSC122758), for 8–10 days. Epithelial clusters appeared and increased during this period, and were picked and embedded into 50 μL Matrigel droplets. Each droplet containing 10–15 epithelial clusters was cultured in HLP medium (minus BMP4 and RA) with saline or 200 ng/mL BDNF (Peprotech, 450-02) for 10–14 days.

### scRNA-seq analysis

#### Processing of sequencing files

The FASTQ files of single-cell libraries generated from Illumina NovaSeq system underwent adaptor index removal by Trim Galore (0.5.0). The clean FASTQ files were aligned to the Hg38 and Mm10 genomes with human and mouse gene annotation based on Gencode v35 and vM21 versions, respectively, by STARsolo function of STAR (2.7.6a).^69^ For details on the quality of reads, please see Supplementary information, Table S9.

#### Cell filtering, clustering, and visualization

Low-quality cells were filtered out by the number of UMIs and total counts on each sample. We used Scrublet (1.0)^70^ to predict and exclude potential multiplets. To minimize batch effects, we defined the major cell clusters for each sample separately. Briefly, we applied Scanpy (1.7.2)^71^ to reduce the dimensionality of cells by PCA and perform clustering and visualization by Leiden and UMAP algorithms.^72^ Cell clusters were assigned to known lung cell types based on cell type-specific markers. See Supplementary information, Table S9 for the details on data quality control.

For the visualization of all scRNA-seq data, we first identified the highly variable TFs (scanpy.pp.highly_variable_genes) and differentially expressed genes (DEGs) (*P* value < 0.001, Wilcoxon rank-sum test) among major cell types. These TFs and DEGs (1144 genes) were used for computing PCA. We next applied FFT-accelerated Interpolation-based t-SNE^73,74^ (FIt-SNE) algorithm to display the layout in two dimensions using the top 10 principal components.

#### Identification of the major cell type markers

All data were normalized using functions scanpy.pp.normalize and scanpy.pp.log1p. DEGs of each major cell type were calculated based on the normalized data (*P* value < 0.01, Wilcoxon rank-sum test, log fold change ≥ 0.25). DEGs were listed in Supplementary information, Table S1.

#### Clustering and defining cell subtypes

Top highly variable genes (HVGs) of each sample were selected and merged. After PCA-based dimensionality reduction, we used harmony to correct batch effects based on top 50 PCs via harmony.run_harmony (max_iter_harmony = 15, max_iter_kmeans = 10) implemented by Python package harmonypy^75^ (0.0.6). Cell clusters were identified using the Leiden algorithm (resolution = 0.5, Scanpy) based on harmony space. Genes with *P* value < 0.01 (Wilcoxon rank-sum test) and gene log fold change ≥ 0.25 for each cluster were selected as DEGs. Clusters sharing top DEGs (*n* = 20) were merged before being classified as cell subtypes. DEGs of cell subtypes can be found in Supplementary information, Table S2.

#### Trajectory inference for lung epithelial cell and SMC development

To map the developmental trajectories of lung epithelial cells, we integrated and analyzed the dataset obtained in this study and the two datasets from 10× Genomics platform: Miller et al.^9^ (ArrayExpress: E-MTAB-8221) of 11.5 weeks, 15.0 weeks, 18.0 weeks, 21.0 weeks and Travaglini et al.^13^ (EGA: EGAS00001004344) of adult lung. The union of TFs within the top 5000 HVGs and top 100 HVGs per dataset (643 genes) were selected for analysis. Next, we applied monocle3^76^ to pre-process data, reduce dimensionality, and visualize trajectory. To discern proximal and distal epithelium lineages, as well as VSMC and ASMC, we computed developmental trajectories utilizing WOT (1.0.8.post2),^20^ an optimal transport-based approach to infer ancestor–descendant relationships between cells across adjacent time points of development. We obtained the cell trajectory scores via tmap_model.ancestors and tmap_model.trajectories implemented in the Python package WOT.

For VSMC and ASMC trajectory inference, the cells of weeks 4–5 from SC_Early with trajectory scores > 0.0001 for VSMC and ASMC were considered as SMC progenitors. The predicted progenitors combined with SMCs were selected to perform trajectory analysis again using Python package harmonyTS (0.1.4).^77^ The developmental pseudotime and branch probability of VSMC and ASMC were obtained via Python package Palantir (1.0.0).^61^ Then, we smoothed the gene expression via R package gam (1.20.1). The heatmaps illustrating the gene expression tendency along the developmental trajectory of ASMC and VSMC were generated by R package ComplexHeatmap (2.14.0) (Fig. 5c). To observe the bias of cell fate at weeks 4, 5, and 6, we estimated the cell fate probability via tmap_model.fates implemented in Python package WOT.

#### TF activity inference

To discover the transcriptional regulators of lung epithelial proximal–distal patterning, we applied pySCENIC (0.11.0)^19^ algorithm to identify significant regulons (NES > 1). We then calculated the TF activity based on gene expression levels of each regulon. Weighted sums of TF activities were calculated based on WOT trajectory scores at each time point. The motifs listed in this study were validated by https://jaspar.genereg.net. For details on the TF list, please see Supplementary information, Tables S3 and S7.

#### RNA velocity analysis

We applied the scVelo^29^ (0.2.3) package to calculate the RNA velocity of epithelial cells at weeks 4, 6 and 8. For each time point, the top 10 PCs of top 3000 HVGs were used as input. For details of these processes, please see the scVelo pipeline (https://scvelo.readthedocs.io/index.html).

#### Comparison of TFs between human and mouse epithelia

We collected the mouse lung epithelial single-cell data from E12.0 to E14.0 with an interval of 0.5 days. Low-quality cells were filtered out by the number of detected genes (< 1800 & > 11,000) and total counts (counts > 100,000). We used Scrublet to predict and exclude potential doublet cells (score > 0.4). Clustering and visualization were implemented by functions scanpy.tl.leiden and scanpy.tl.umap, respectively. The *Nkx2-1* (the core TF for lung epithelium development) and *Cdh1* (a universal marker for epithelial cells) positive populations were selected as the mouse lung epithelial cells. *Sox2*/*Sox21*/*Klf5-* and *Sox9*/*Gata6*/*Etv5*-expressing cells were annotated as proximal and distal epithelial cells, respectively.

Human–mouse orthologous genes were used for comparison (Human and Mouse Homologs from MGI database, https://www.informatics.jax.org/homology.shtml). We calculated the differentially expressed TFs in human and mouse (*P* value < 0.01, *t*-test), respectively. The TFs were divided into 5 categories: proximal shared, distal shared, human-specific, mouse-specific and human–mouse inverse. The TF list can be found in Supplementary information, Table S5.

#### Comparison of human and mouse stromal cells

To compare mesenchymal lineages between human and mouse, we integrated and analyzed stromal cells from this study and the Goodwin et al.^47^ (GEO: GSE153069) mouse dataset from the 10× Genomics platform. Orthologous genes in human and mouse were extracted for comparison. Then, we calculated species-specific and cell type-specific DEGs across human and mouse stromal cell types at 6–7 weeks and E11.5 (*P* value < 0.01, Wilcoxon rank-sum test). These DEG sets included ‘Common markers for stromal cells’, ‘Shared DEGs’, ‘Human SC_BDNF^+^ specific’, ‘Mouse mesothelium-specific’ and ‘Mouse sub-meso-specific’. For details on DEGs, please see Supplementary information, Table S10.

#### Ligand–receptor interaction analysis

We integrated the ligand–receptor databases, cellphoneDB^78^ and intact (https://www.ebi.ac.uk/intact). The product of the expression percentage (nUMI > 0) of a given ligand and its receptor from the corresponding provider and recipient cells was calculated as the ligand–receptor score. To calculate the significance of the interaction for each ligand–receptor pair, cell type labels were permuted 1000 times to generate the background distribution of ligand–receptor scores (standardized to mean of 0 and standard deviation of 1). *P* value < 0.001 (U-test) was considered significant. For details on the ligand–receptor list, please see Supplementary information, Table S6.

### Spatial transcriptomic analysis

#### Processing of sequencing files

The FASTQ files of the spatial transcriptomic library was generated by MGISEQ2000. The FASTQ files were aligned to the hg38 genome with human gene annotation based on Gencode v32 using spaceranger (2.0.0). The serial number of the Visum slide is V11T16-101, the slide file can be downloaded from https://support.10xgenomics.com/spatial-gene-expression/software/pipelines/latest/using/slidefile-download. For the details of data quality, please see Supplementary information, Table S9.

#### Basic analysis and visualization of the Visium data

Low-quality spots were filtered out if the number of detected genes < 2000. The spatial data were normalized via functions scanpy.pp.normalize and scanpy.pp.log1p. Spatial visualization was performed via function scanpy.pl.spatial.

#### Mapping single-cell data to spatial transcriptomic data

We used Tangram^35^ (1.0.3) to map the annotated scRNA-seq data to the spatial spots. To match these two omics, we selected the 6-week data for mapping, using unnormalized UMI counts as input. For the spatial data, we calculated the Moran’I (*I*, a measure of spatial autocorrelation) for each gene. Genes with absolute *I* greater than 0.05 were selected as spatially variable genes (SVGs), as calculated by squipy.gr.spatial_autocorr function of Python package squidpy^79^ (1.2.3). A total of 2450 genes of SVGs and HVGs (from single-cell data) were used for mapping, resulting in a probability matrix of the assignment of each cell to all spots. To further access the proportion of cell types in each spot, we summed the probability of each single-cell-annotated cell type. For the details of mapping probability, please see Supplementary information, Table S11.

#### The adjacent score for stromal cells and epithelial cells

We first identified spots surrounding the proximal or distal epithelium region. These spots, as well as those in the epithelial region, were defined as proximal- or distal-associated spots, respectively. To reduce the integration noise between single-cell and spatial data, cell types with a proportion less than 0.4 were excluded in each selected spot. The adjacent score of each cell type to the epithelium was calculated by summing their proportions in proximal- or distal-associated spots. The scores were displayed by Sankey diagram via sankeyNetwork function of R package networkD3 (0.4). The information can be found in Supplementary information, Table S11.

#### Evaluating the score of cellular interactions in Visium slides

We first assigned each cell to a spot based on the maximum mapping probability from Tangram, defined as a recipient cell (expressing receptors), surrounded by provider cells (expressing ligands) in the neighboring spots. Their ligand–receptor scores and *P* values were calculated as described in the Ligand–receptor interaction analysis section. The ligand–receptor score between distal epithelium and SC_BDNF^+^ were shown in Supplementary information, Fig. S7c.

## Supplementary information

Supplementary information, Fig. S1

Supplementary information, Fig. S2

Supplementary information, Fig. S3

Supplementary information, Fig. S4

Supplementary information, Fig. S5

Supplementary information, Fig. S6

Supplementary information, Fig. S7

Supplementary information, Fig. S8

Supplementary information, Fig. S9

Supplementary information, Fig. S10

Supplementary information, Table S1

Supplementary information, Table S2

Supplementary information, Table S3

Supplementary information, Table S4

Supplementary information, Table S5

Supplementary information, Table S6

Supplementary information, Table S7

Supplementary information, Table S8

Supplementary information, Table S9

Supplementary information, Table S10

Supplementary information, Table S11

## Data Availability

The scRNA-seq and spatial transcriptome data reported in this paper have been deposited in the OMIX, China National Center for Bioinformation/Beijing Institute of Genomics, Chinese Academy of Sciences (https://ngdc.cncb.ac.cn/omix: accession number OMIX003147). The raw data reported in this study can be requested from the corresponding author (chen_jiekai@gibh.ac.cn).
