# Supplementary material for: Single-cell RNA sequencing reveals the developmental program underlying proximal–distal patterning of the human lung at the embryonic stage
Source: Cell Res. 2023 Apr 21;33(6):421–33. doi: 10.1038/s41422-023-00802-6 (PMC10119843; doi:10.1038/s41422-023-00802-6)
Supplement: Supplementary file 1 — Supplementary information, Fig. S1 [file 41422_2023_802_MOESM1_ESM.pdf]

Supplementary information, Fig. S1

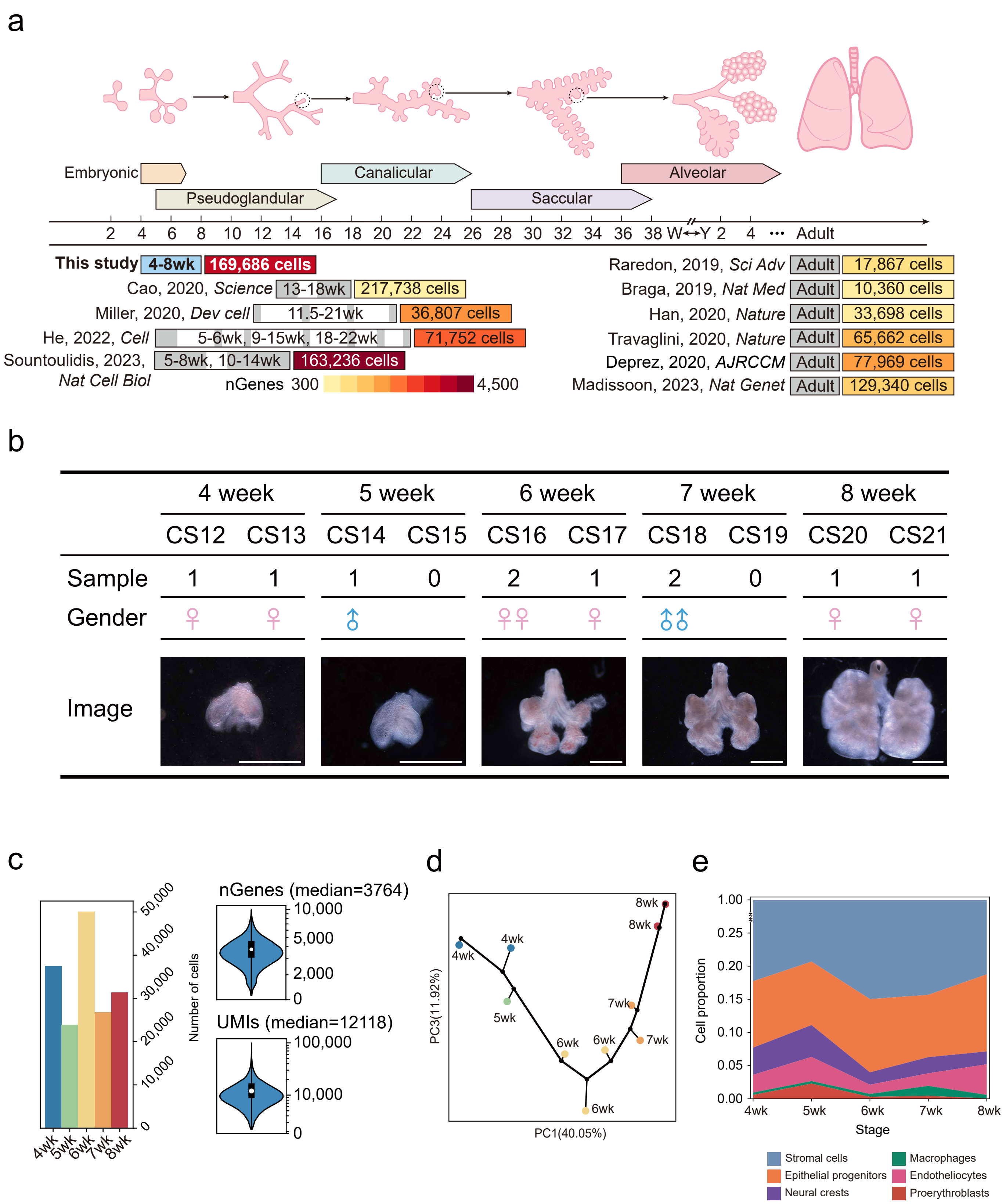

Fig. S1 Tissue images and data quality.

(a) Comparison of single cell studies for human lung development.

(b) Representative human embryonic lung image, Carnegie stage, and sample size of scRNA-seq experiments. Scale bar, 1mm.

(c) Histograms showing the number of cells sequenced at each timepoint. The distribution of detected genes (nGenes) and counts (UMIs) across all samples were shown in violin plots.

(d) PCA of pseudo-bulk RNA-seq profiles demonstrating a concordance collection time of samples along the PC components.

(e) Stack plot showing the proportion of cell types over time.
