# Supplementary material for: Single-cell RNA sequencing reveals the developmental program underlying proximal–distal patterning of the human lung at the embryonic stage
Source: Cell Res. 2023 Apr 21;33(6):421–33. doi: 10.1038/s41422-023-00802-6 (PMC10119843; doi:10.1038/s41422-023-00802-6)
Supplement: Supplementary file 2 — Supplementary information, Fig. S2 [file 41422_2023_802_MOESM2_ESM.pdf]

Supplementary information, Fig. S2

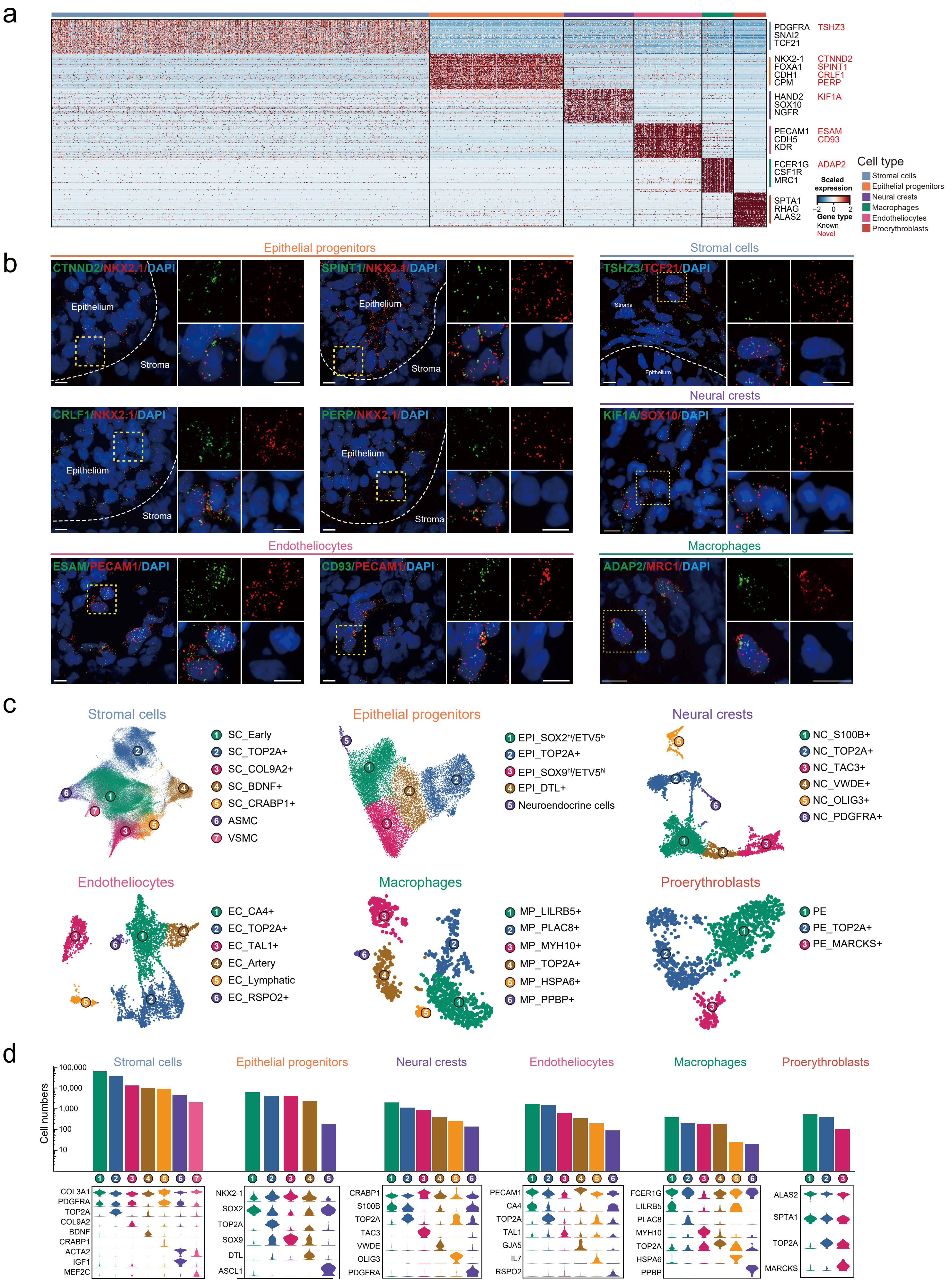

Fig. S2 Human embryonic lung cell atlas.

(a) Heatmap showed scaled expression of differentially expressed genes (DEGs) among six major cell types (p-value<0.01, Wilcoxon rank-sum test). Genes marked in black are the known cell type markers, in red are the newly identified markers in this study.

(b) smiFISH staining in 8 weeks' lung tissue showing epithelial (i.e., *NKX2-1*, *CTNND2*, *SPINT1*, *CRLF1*, *PERP*), stromal (i.e., *TSHZ3*, *TCF21*), neural crest (i.e., *KIF1A*, *SOX10*), endothelial (i.e., *ESAM*, *PECAM1*), and macrophage (i.e., *ADAP2*, *MRC1*) markers. Data are representative of at least two independent smiFISH experiments. Scale bar, 10μm.

(c) Further clustering of the six major cell types, yielding 33 cell subtypes. Cell subtypes of each major cell types are projected on UMAP layout, respectively.

(d) Bar plot showing the log10-transformed cell number of 33 cell subtypes (upper). Violin plot showing the distribution of shared and specific markers of these cell subtypes (lower).
