# Supplementary material for: Single-cell RNA sequencing reveals the developmental program underlying proximal–distal patterning of the human lung at the embryonic stage
Source: Cell Res. 2023 Apr 21;33(6):421–33. doi: 10.1038/s41422-023-00802-6 (PMC10119843; doi:10.1038/s41422-023-00802-6)
Supplement: Supplementary file 3 — Supplementary information, Fig. S3 [file 41422_2023_802_MOESM3_ESM.pdf]

Supplementary information, Fig. S3

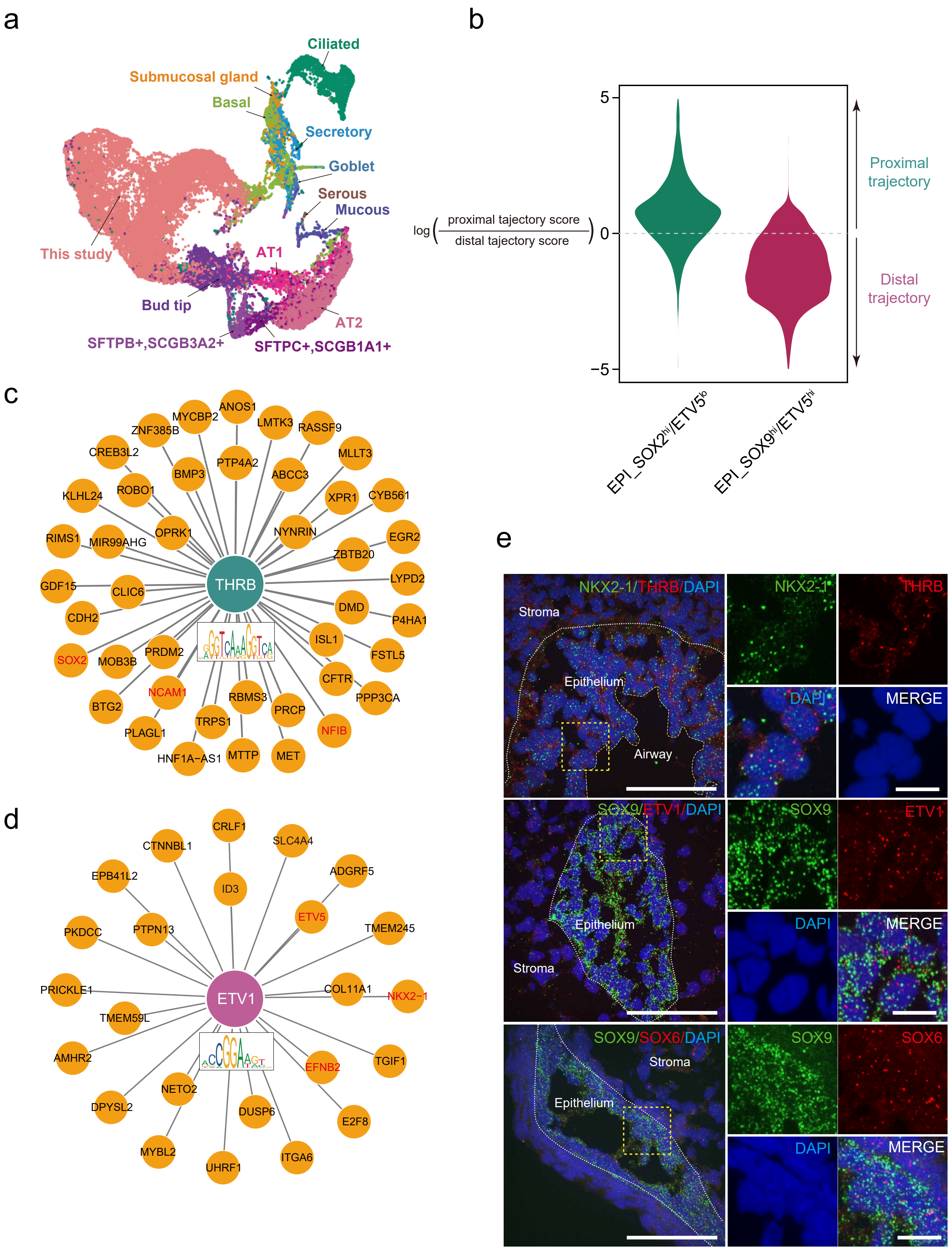

Fig. S3 Development of human embryonic lung epithelium.

(a) UMAP layout showing the annotation of lung epithelial cell types.

(b) The predicted proximal and distal cell fates of EPI_SOX2^{hi}/ETV5^{lo} and EPI_SOX9^{hi}/ETV5^{hi} epithelial cells based on more mature proximal and distal cell types from weeks 11.5 - 21 of published dataset[9]. The trajectory inference was performed using Waddington-OT (WOT) (see Materials and Methods).

(c-d) Network showing the SCENIC predicted target genes of *THRB* **(c)** and *ETV1* **(d)**. The genes marked in red are contributive to proximal epithelium reported in previous studies. The motifs listed in this study are validated from https://jaspar.genereg.net.

(e) smiFISH staining the expression of *THRB* (red) and *NKX2-1* (green) in proximal epithelium, the expression of *ETV1* (red)/*SOX6* (red) and *SOX9* (green) in distal epithelium in the lung at week 8. Data are representative of at least two independent smiFISH experiments. Scale bar: 50 μm (long), 10μm (short).
