# Supplementary material for: Single-cell RNA sequencing reveals the developmental program underlying proximal–distal patterning of the human lung at the embryonic stage
Source: Cell Res. 2023 Apr 21;33(6):421–33. doi: 10.1038/s41422-023-00802-6 (PMC10119843; doi:10.1038/s41422-023-00802-6)
Supplement: Supplementary file 4 — Supplementary information, Fig. S4 [file 41422_2023_802_MOESM4_ESM.pdf]

Supplementary information, Fig. S4

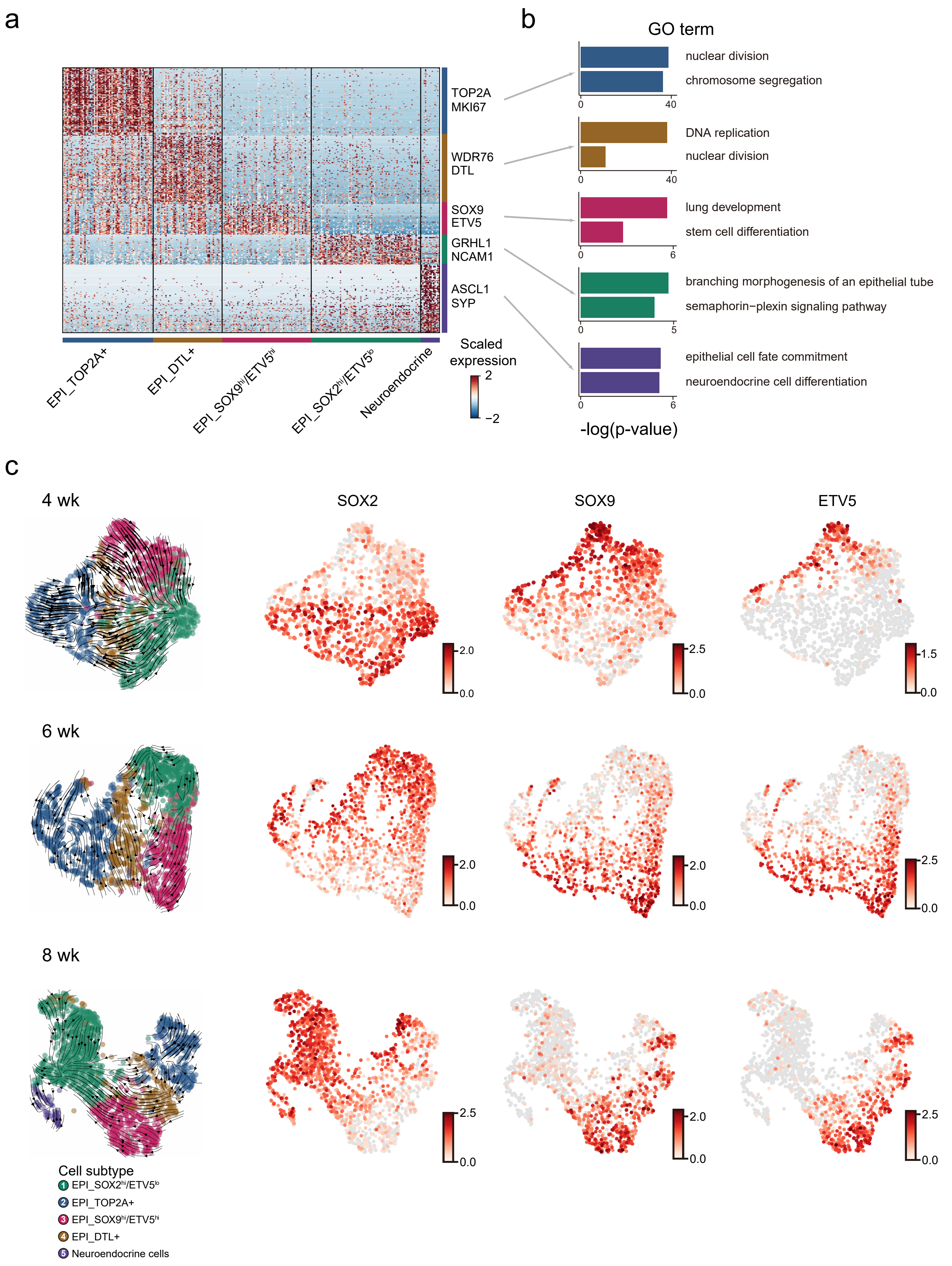

Fig. S4 Heterogeneity of lung epithelial cells.

(a) Heatmap showing differentially expressed genes of 5 epithelial cell subtypes, with representative genes listing on the right side.

(b) Bar plots showing the Gene Ontology (GO) terms of 5 epithelial cell subtypes. GO enrichment was performed using clusterProfiler.

(c) UMAP projecting RNA velocity and the expression of *SOX2*/*SOX9*/*ETV5* at weeks 4, 6, 8. The cell subtypes were annotated by their markers corresponding to **Fig. S2c.**
