# Supplementary material for: Single-cell RNA sequencing reveals the developmental program underlying proximal–distal patterning of the human lung at the embryonic stage
Source: Cell Res. 2023 Apr 21;33(6):421–33. doi: 10.1038/s41422-023-00802-6 (PMC10119843; doi:10.1038/s41422-023-00802-6)
Supplement: Supplementary file 5 — Supplementary information, Fig. S5 [file 41422_2023_802_MOESM5_ESM.pdf]

Supplementary information, Fig. S5

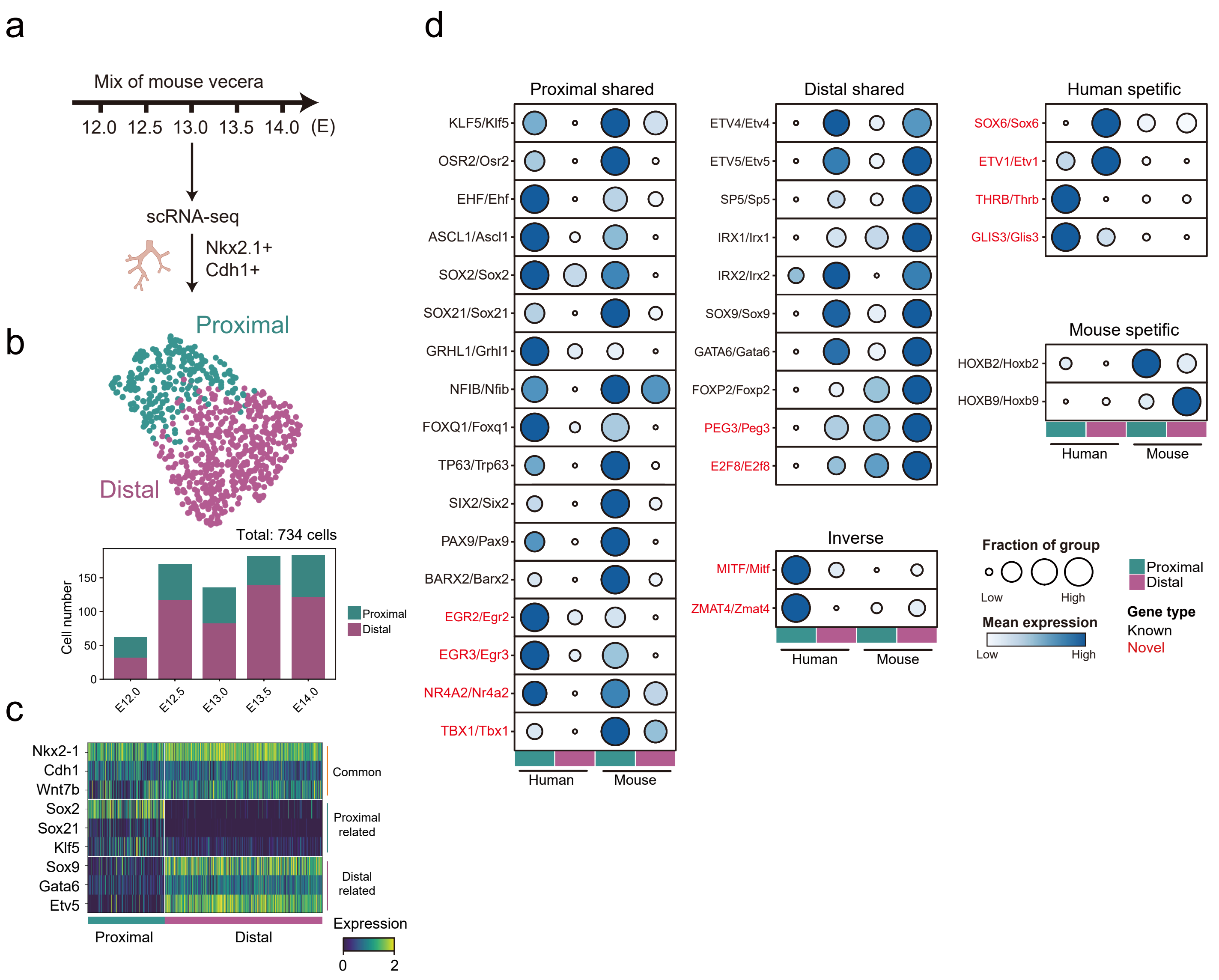

Fig. S5 Comparison of TF expression between human and mouse.

(a-c) Single cell data of mouse lung epithelium was obtained from E12.0 to E14.0 embryos with an interval of 0.5 embryonic day. *Nkx2.1*+ and *Cdh1*+ population were selected as lung epithelial cells for analysis **(a)**. UMAP showing the proximal and distal epithelium labels, bar plot showing the number of proximal and distal epithelium per time point **(b)**. Heatmap showing the expression of the well-known epithelial markers **(c)**.

(d) Dot plot showing similarities and differences of TF between human and mouse epithelium. The patterns of TF expression were divided into proximal shared, distal shared, human-mouse inverse, human-specific and mouse-specific. The size and color of each dot represented the expression percentage and average expression level of one marker gene within one group, respectively. Genes marked in red are the newly identified markers in this study.
