# Supplementary material for: Single-cell RNA sequencing reveals the developmental program underlying proximal–distal patterning of the human lung at the embryonic stage
Source: Cell Res. 2023 Apr 21;33(6):421–33. doi: 10.1038/s41422-023-00802-6 (PMC10119843; doi:10.1038/s41422-023-00802-6)
Supplement: Supplementary file 6 — Supplementary information, Fig. S6 [file 41422_2023_802_MOESM6_ESM.pdf]

Supplementary information, Fig. S6

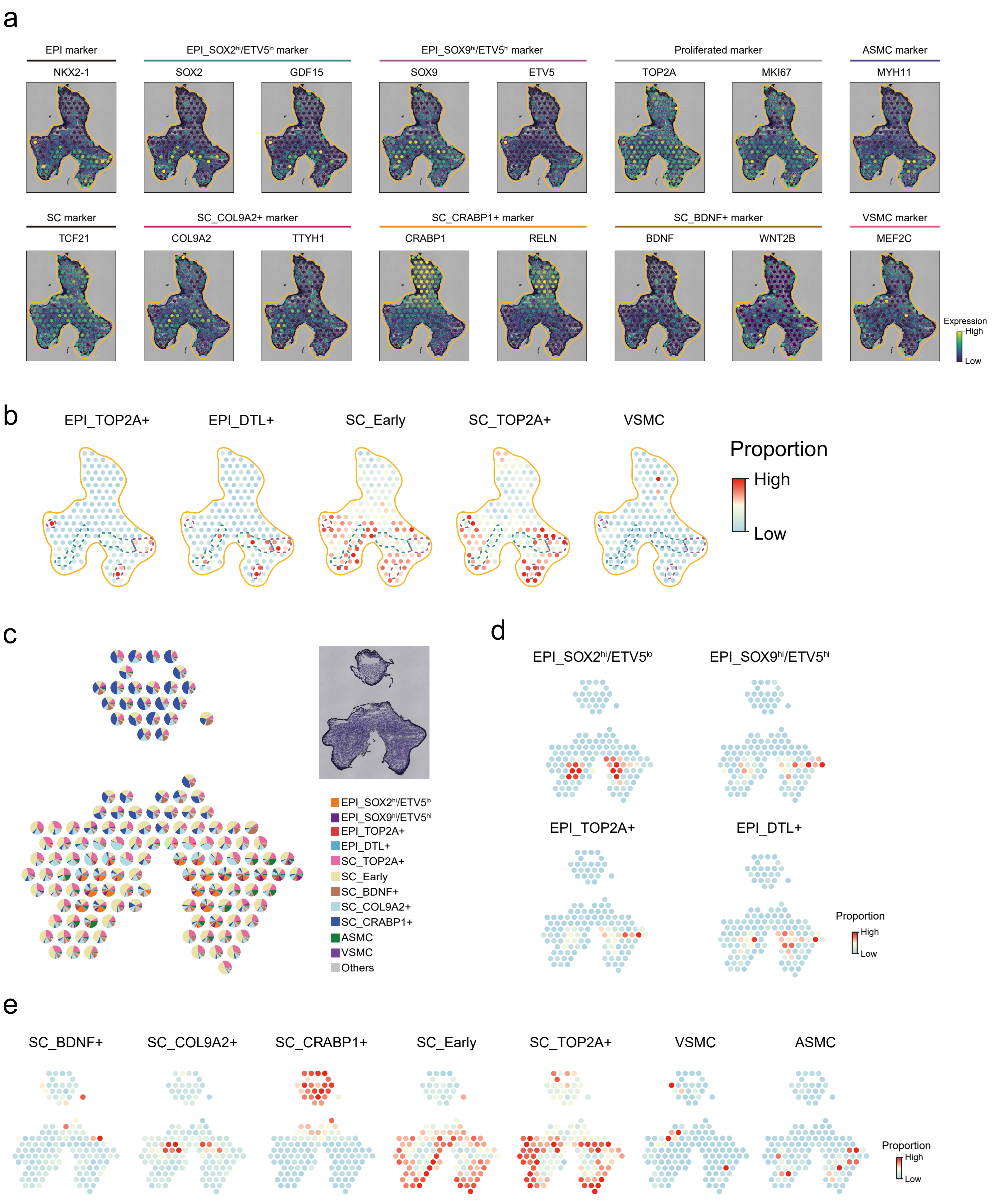

Fig. S6 Multiple epithelial and stromal cell types exhibit proximal-distal spatial heterogeneity.

(a) Dot plot showing the gene expression of epithelial cell types and stromal cell type on the 10x Visium spots.

(b) Dot plots showing the proportion of two epithelial cell types and three stromal cell types mapped to 10x Visium spots, respectively. The orange solid line, green dashed line and magenta dashed line indicate the outline of lung, proximal epithelium and distal epithelium, respectively, consistent with the illustration of **Fig. 3e.**

(c) Pie plots showing the proportion of cell types mapped to each 10x Visium spot from single-cell transcriptome data (left) of another tissue section.

(d) Dot plots showing the proportion of four epithelial cell types mapped to 10x Visium spots.

(e) Dot plots showing the proportion of seven stromal cell types mapped to 10x Visium spots.
