# Supplementary material for: Single-cell RNA sequencing reveals the developmental program underlying proximal–distal patterning of the human lung at the embryonic stage
Source: Cell Res. 2023 Apr 21;33(6):421–33. doi: 10.1038/s41422-023-00802-6 (PMC10119843; doi:10.1038/s41422-023-00802-6)
Supplement: Supplementary file 7 — Supplementary information, Fig. S7 [file 41422_2023_802_MOESM7_ESM.pdf]

Supplementary information, Fig. S7

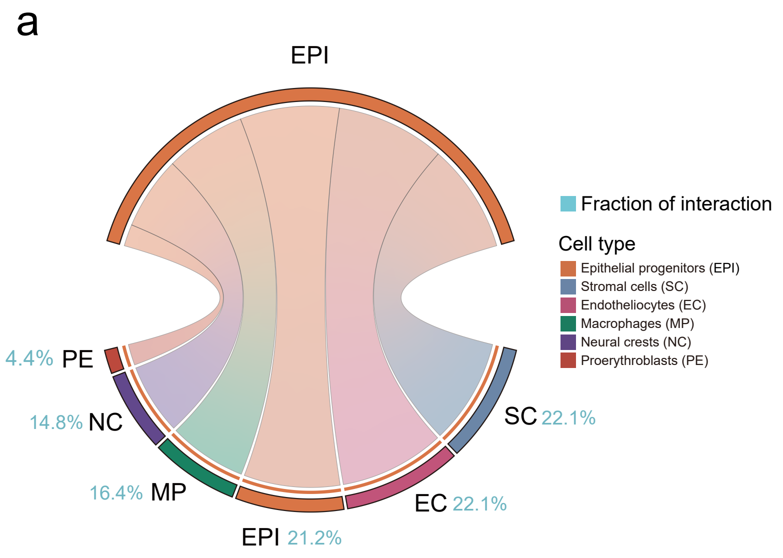

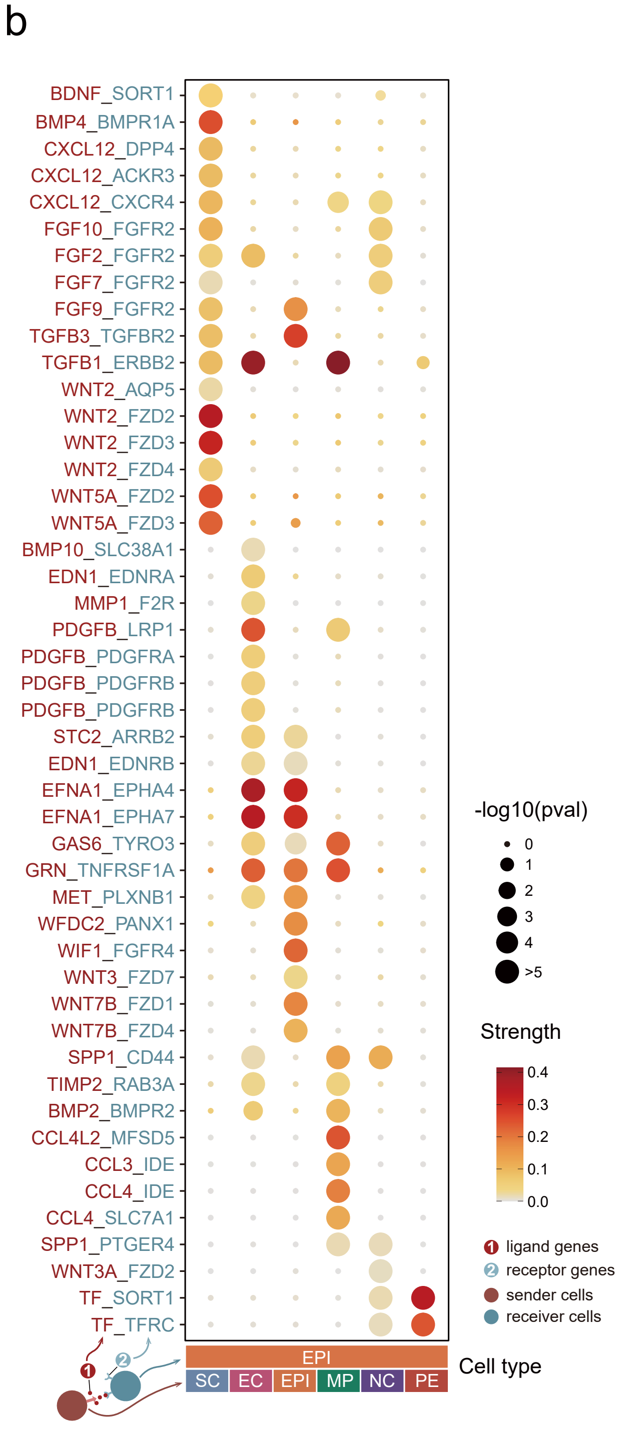

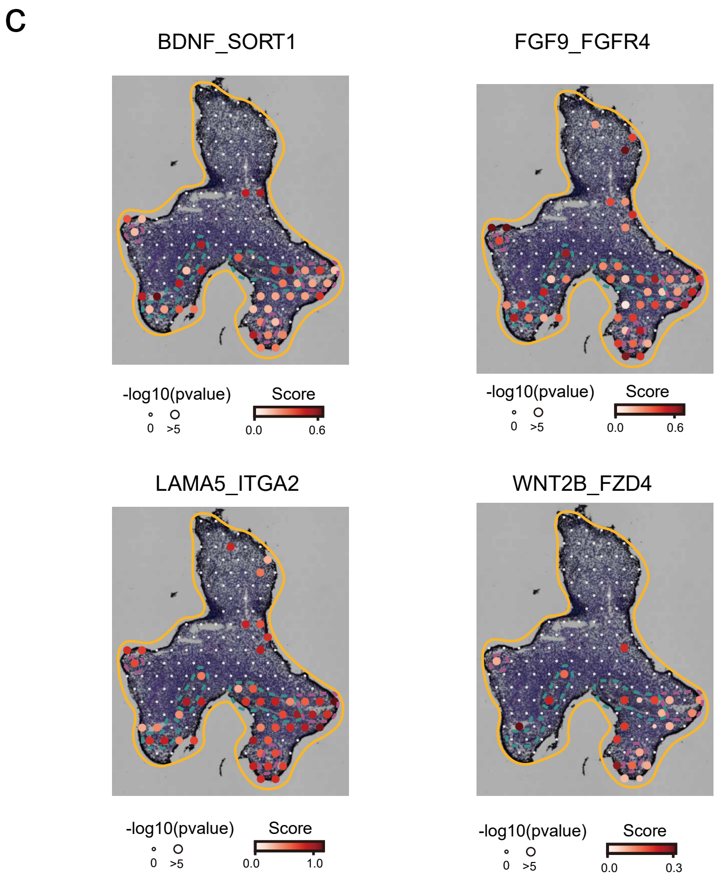

Fig. S7 Intercellular interactions around epithelial cells in human embryonic lung.

(a) Circos plot showing summary of intercellular interactions between epithelial cells and six major cell types. The percentages colored in light blue represented the proportion of ligand-receptor interactions. Abbreviations: EPI, Epithelial cells; SC, Stromal cells; EC, Endotheliocytes; MP, Macrophages; NC, Neural crests; PE, Proerythroblasts.

(b) Dot plot showing the strength of ligand-receptor interactions of six major cell types around epithelial cells, with epithelial cells as the receptor cell types and the other six cell types as ligand cell types (including epithelial cell). The size of dot represented -log10(p-value) (U-test). The color of dot represented interaction score. Genes in blue represented receptor genes, in red represented ligand genes. Color bars of cell types were related to **(a)**.

(c) Dot plot showing the strength of ligand-receptor interactions of SC_BDNF+ and EPI_SOX9^{hi}/ETV5^{hi} (see Materials and Methods) on 10x Visium spot. The size and color of each dot represent the -log10(p-value) (U-test) and interaction score, respectively.
