# Supplementary material for: Single-cell RNA sequencing reveals the developmental program underlying proximal–distal patterning of the human lung at the embryonic stage
Source: Cell Res. 2023 Apr 21;33(6):421–33. doi: 10.1038/s41422-023-00802-6 (PMC10119843; doi:10.1038/s41422-023-00802-6)
Supplement: Supplementary file 8 — Supplementary information, Fig. S8 [file 41422_2023_802_MOESM8_ESM.pdf]

# Supplementary information, Fig. S8

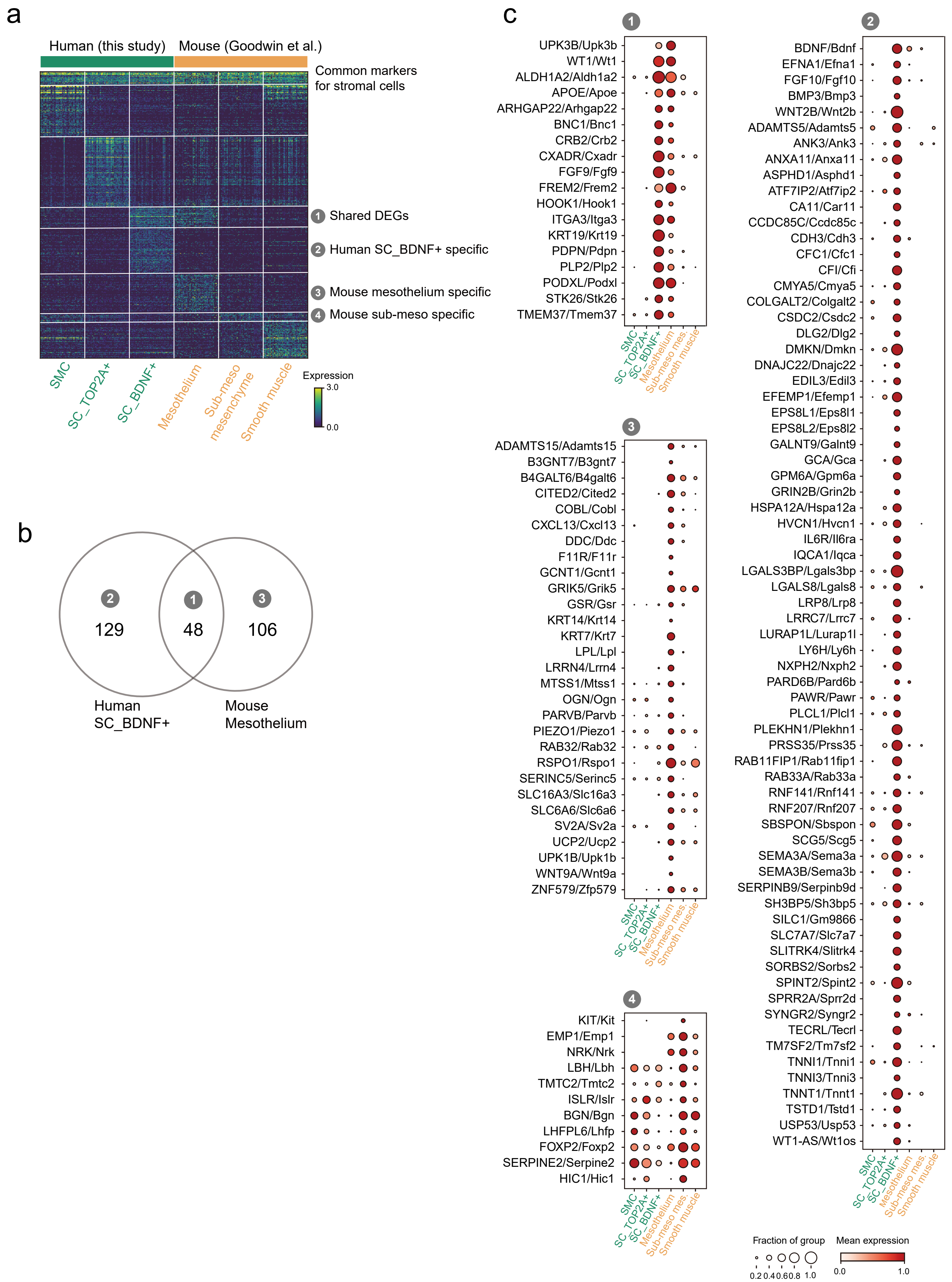

**Fig. S8 Comparison of the human stromal cells and mouse stromal cells.**

**(a)** Heatmap showing the DEGs between human stromal cells (green bar) and mouse stromal cells (orange bar).

**(b)** Venn plot showing the shared and specific DEGs between Human SC_BDNF+ and Mouse mesothelium.

**(c)** Dot plots showing the gene expression of 'Shared DEGs', 'Human SC_BDNF+ specific', 'Mouse mesothelium specific' and 'Mouse sub-meso specific' between human stromal cells (green) and mouse stromal cells (orange), respectively. The size and color of each dot represent the expression percentage and average expression level of one marker gene within one cell type, respectively.
