# Supplementary material for: Single-cell RNA sequencing reveals the developmental program underlying proximal–distal patterning of the human lung at the embryonic stage
Source: Cell Res. 2023 Apr 21;33(6):421–33. doi: 10.1038/s41422-023-00802-6 (PMC10119843; doi:10.1038/s41422-023-00802-6)
Supplement: Supplementary file 9 — Supplementary information, Fig. S9 [file 41422_2023_802_MOESM9_ESM.pdf]

Supplementary information, Fig. S9

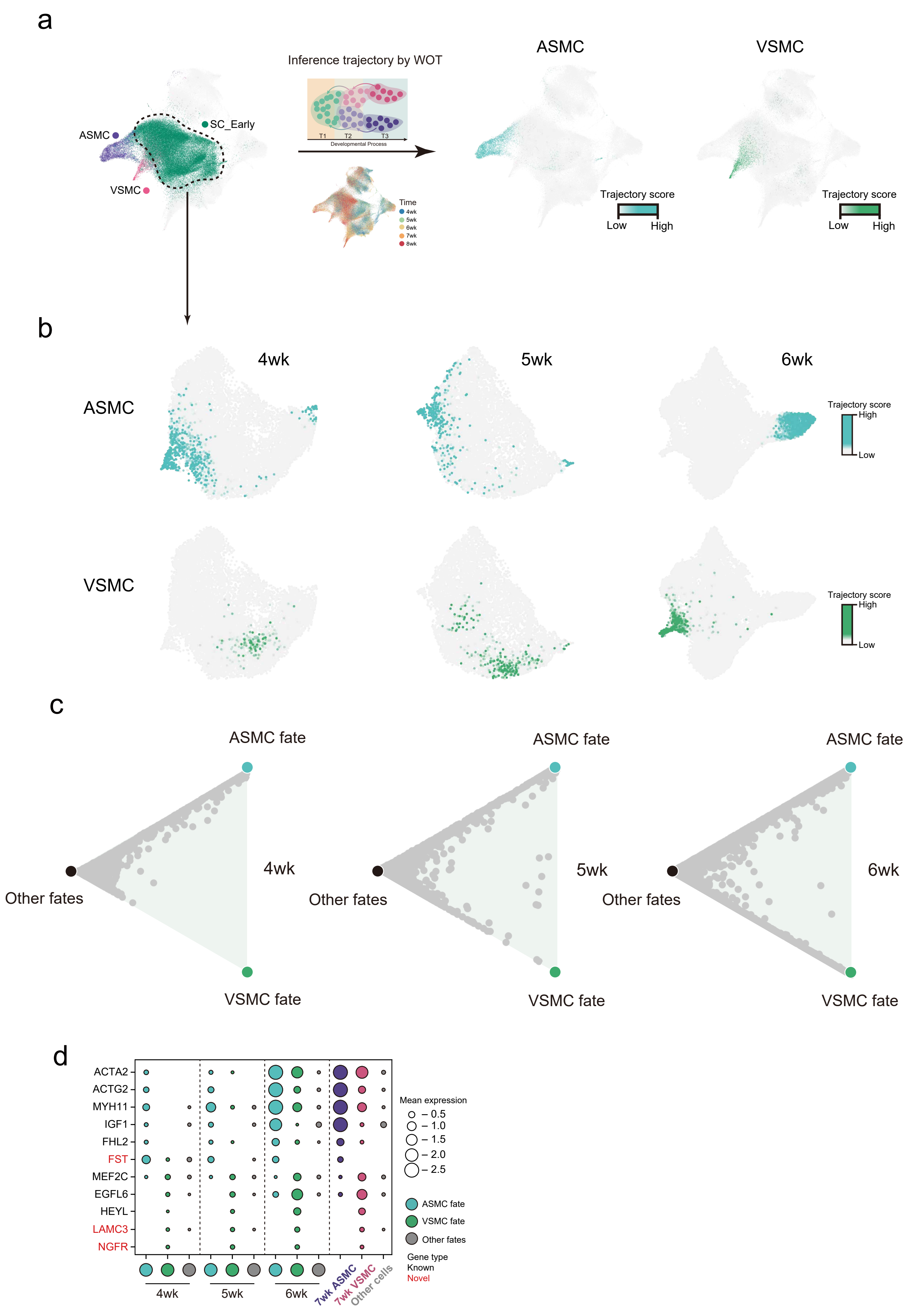

Fig. S9 Discerning VSMC and ASMC progenitors.

(a) UMAP layout showing stromal cell subtypes (SC_Early, VSMC and ASMC). WOT was used to predict progenitors and descendants of ASMC and VSMC, indicated by trajectory score. The higher the score of a cell, the more likely the cell is ASMC (blue) or VSMC (green).

(b) UMAPs showing the VSMC and ASMC trajectory score in stromal cell subtypes. SC_Early during weeks 4-6. Colors indicated the trajectory score of ASMC and VSMC.

(c) Fate matrix of ASMC and VSMC of weeks 4, 5 and 6. Cells with fate probability were calculated via tmap_model.fates implemented in WOT package (see Materials and Methods).

(d) Dot plot showing the weighted-sum expression of VSMC and ASMC markers using trajectory score of each time point.
