# Supplementary material for: Single-cell RNA sequencing reveals the developmental program underlying proximal–distal patterning of the human lung at the embryonic stage
Source: Cell Res. 2023 Apr 21;33(6):421–33. doi: 10.1038/s41422-023-00802-6 (PMC10119843; doi:10.1038/s41422-023-00802-6)
Supplement: Supplementary file 10 — Supplementary information, Fig. S10 [file 41422_2023_802_MOESM10_ESM.pdf]

Supplementary information, Fig. S10

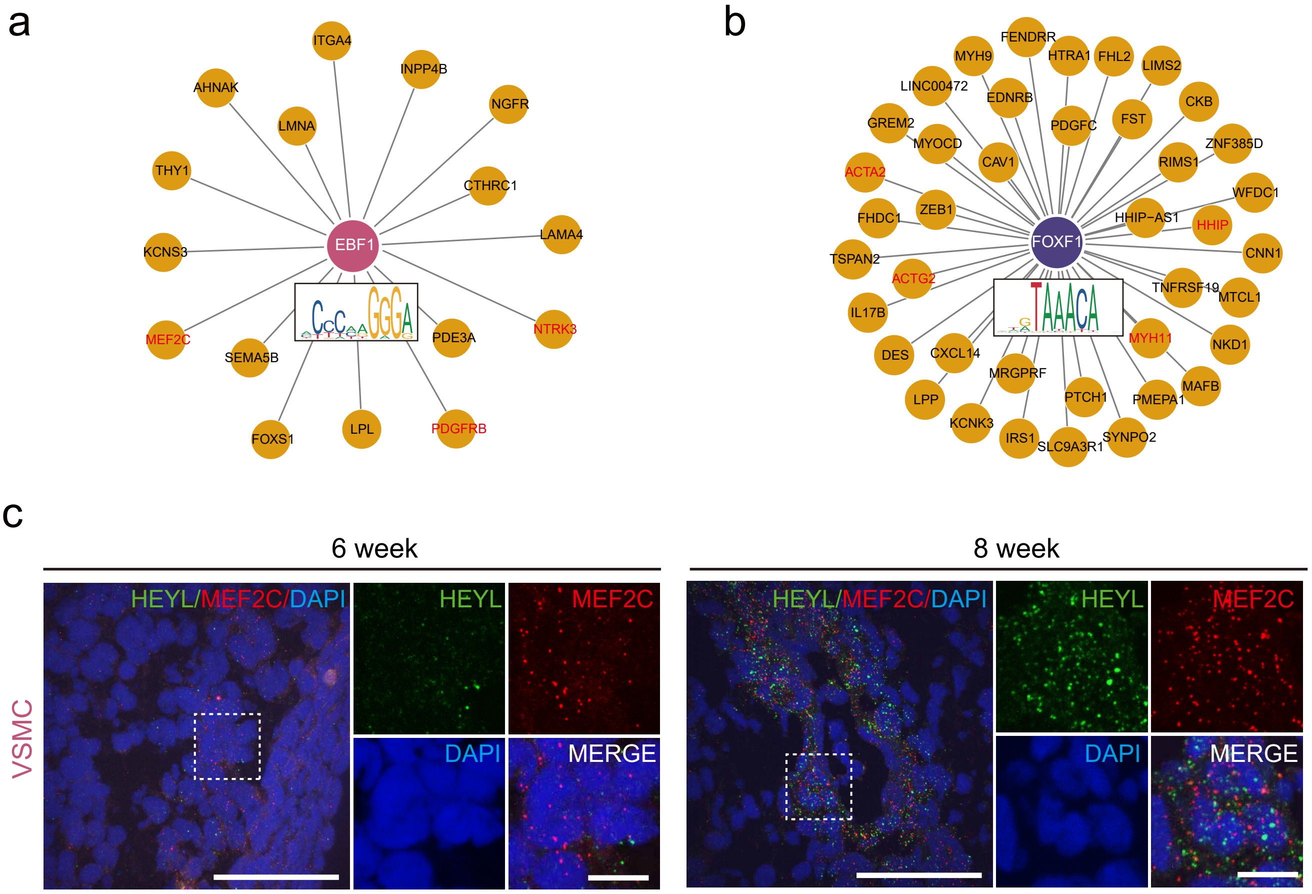

Fig. S10 Transcription factors regulate the development of VSMC and ASMC.

(a-b) Regulon networks of *EBF1* and *FOXF1* were described in **(a)** and **(b)**. The target genes marked in red are reported in previous studies. The motifs listed in this study are validated from https://jaspar.genereg.net.

(c) smiFISH staining the expression of *HEYL* (green) and *MEF2C* (red) in a group of VSMC in the lung at week 6 and 8. Data are representative of at least two independent smiFISH experiments. Scale bar: 50 μm (long), 10 μm (short).
